# Supplementary material for: RS-FISH: precise, interactive, fast, and scalable FISH spot detection
Source: Nat Methods. 2022 Nov 17;19(12):1563–7. doi: 10.1038/s41592-022-01669-y (PMC9718671; doi:10.1038/s41592-022-01669-y)
Supplement: Supplementary file 1 — Supplementary Notes 1–6, Supplementary Figures SN1–12, and Supplementary Table 3 [file 41592_2022_1669_MOESM1_ESM.pdf]

nature methods

Brief Communication

https://doi.org/10.1038/s41592-022-01669-y

RS-FISH: precise, interactive, fast, and scalable FISH spot detection

In the format provided by the authors and unedited

Supplementary Notes:

1. Derivation of the three-dimensional Radial Symmetry localization [link]
2. Derivation for supporting axis-aligned ellipsoid (non-radial) objects [link]
3. Computing the global scale factor [link]
4. Accuracy benchmarking RS-FISH against commonly used tools [link]
5. Computation time benchmarking [link]
6. Benchmarking on real data [link]
7. Close spots analysis [link]
8. Choosing the right parameters for RS-FISH [link]
9. Tutorial RS-FISH [link]
10. Batch processing and macro tutorial [link]
11. Distributed processing using RS-FISH-Spark [link]
12. Filtering detections using binary masks [link]
13. smFISH protocol for *C. elegans* [link]
14. smFISH protocol for mouse ES cells using cytospin [link]
15. smFISH protocol for cleared whole-mount adult Drosophila brains [link]
16. smFISH protocol for Kuramochi cells [link]

Supplementary Tables:

1. Comparison of localization performance on simulated data [link]
2. Comparison of execution time of real 3D datasets [link]
3. Information on datasets used in this publication [link]
4. Results table of simulated data [link]
5. Results table of real image benchmarking [link]
6. Results table of close spot detections [link]

1. Derivation of the three-dimensional Radial Symmetry Localization

(and obvious extension to the n-dimensional case)

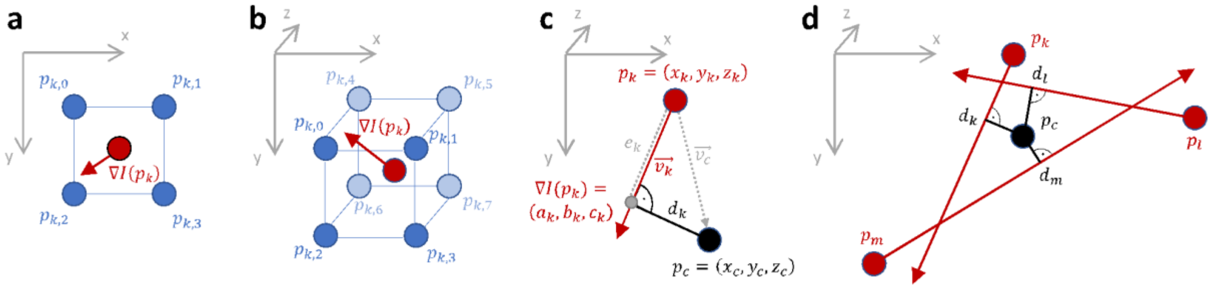

Figure SN1.1: *Concept of 3D Radial Symmetry, derived from Parthasarathy [1].* (**a**) *illustrates for the two-dimensional case the concept of the intensity gradient $\nabla I(p)$ at the half-pixel offset locations p, computed from four intensity values $I(p)$ at locations $\{p_0, p_1, p_2, p_3\}$ that intersects with the intensity maximum.* (**b**) *generalizes the concept to three dimensions, which requires eight intensity values $I(p)$ at locations $\{p_0, \ldots, p_7\}$.* (**c**) *Parthasarathy defined the center point $p_c = (x_c, y_c, z_c)$ and the shortest distance to $\nabla I(p)$ as d_k, which directly extends to 3D.* (**d**) *Illustrates that minimizing $\sum_i d_i$ yields the common intersection point p_c.*

Our extension of Radial Symmetry from two dimensions (2D) to three dimensions (3D) requires adjustments of gradient computation and is based on a new derivation that can scale to the n-dimensional case. Although we only show the 3D case here, we suggest how to straightforwardly extend these concepts to higher dimensions. General concepts are illustrated in **Fig SN1.1**.

First, image gradients $\nabla I(p)$ are computed, which are located on a half-pixel offset grid (**Fig SN1.1a,b**). Parthasarathy[1] suggested using the Roberts cross operator, which, however, cannot be straightforwardly extended to 3D. Instead, we use separable computation of image gradients for the 2D case:

$$\nabla I(p) = \begin{bmatrix} \frac{\partial I}{\partial x}(p) \\ \frac{\partial I}{\partial y}(p) \end{bmatrix} = \begin{bmatrix} \frac{I(p_1)+I(p_3)-I(p_0)-I(p_2)}{2} \\ \frac{I(p_2)+I(p_3)-I(p_0)-I(p_1)}{2} \end{bmatrix}$$

and the 3D case:

$$\nabla I(p) = \begin{bmatrix} \frac{\partial I}{\partial x}(p) \\ \frac{\partial I}{\partial y}(p) \\ \frac{\partial I}{\partial z}(p) \end{bmatrix} = \begin{bmatrix} \frac{I(p_1)+I(p_3)+I(p_5)+I(p_7)-I(p_0)-I(p_2)-I(p_4)-I(p_6)}{4} \\ \frac{I(p_2)+I(p_3)+I(p_6)+I(p_7)-I(p_0)-I(p_1)-I(p_4)-I(p_5)}{4} \\ \frac{I(p_4)+I(p_5)+I(p_6)+I(p_7)-I(p_0)-I(p_1)-I(p_2)-I(p_3)}{4} \end{bmatrix}$$

Due to the separable nature of the computation, it can be easily extended to higher dimensions.

Using these image gradients, it is possible to compute their 3D intersection point $p_c = (x_c, y_c, z_c)$. In contrast to the original 2D radial symmetry, we derive the formula for computing the shortest distance d_k between p_c and the vector $\overrightarrow{v_k}$ defined by the location $p_k = (x_k, y_k, z_k)$ and the gradient $\nabla I(p_k)$ using vector algebra as it extends to higher dimensions (**Fig SN1.1c**). Therefore, we compute the dot product between the vector $\overrightarrow{v_c} = p_c - p_k$ and $\overrightarrow{v_k} = p_k + \dot{\nabla} I(p_k)$ which projects $\overrightarrow{v_c}$ onto $\overrightarrow{v_k}$ computing the helper distance e_k:

$$e_k = |\overrightarrow{v_c}| \cdot \frac{\overrightarrow{v_k}}{|\overrightarrow{v_k}|}$$

From that, one can directly compute

$$d_k = |p_c - e_k \overrightarrow{v_k}|$$

Replacing the actual coordinates and reformulating the above equation yields for the 3D case:

$$\Delta x = x_k - x_c$$
$$\Delta y = y_k - y_c$$
$$\Delta z = z_k - z_c$$
$$\alpha = a_k * \Delta x + b_k * \Delta y + c_k * \Delta z$$
$$d_k^2 = \Delta x^2 + \Delta y^2 + \Delta z^2 - \frac{\alpha^2}{a_k^2 + b_k^2 + c_k^2}$$

for which the pattern for an extension to the n-dimensional case becomes obvious when comparing it to the 2D case:

$$\Delta x = x_k - x_c$$
$$\Delta y = y_k - y_c$$
$$\alpha = a_k * \Delta x + b_k * \Delta y$$
$$d_k^2 = \Delta x^2 + \Delta y^2 - \frac{\alpha^2}{a_k^2 + b_k^2}$$

We then derived the least-squares fit for identifying the common intersection point $p^c = (x_c, y_c, z_c)$ of all gradients by minimizing (**Fig SN1.1d**):

$$\chi^2 \equiv \sum\nolimits_k d_k^2 w_k$$

where w_k denotes optional weights for each gradient $\nabla I(p^k)$. Derivations for the 3D case:

$$\frac{\partial \chi^2}{\partial x_c} = 0, \qquad \frac{\partial \chi^2}{\partial y_c} = 0, \qquad \frac{\partial \chi^2}{\partial z_c} = 0$$

yields a set of linear equations that can be expressed in matrix form $\Delta * p_c = \Theta$

$$m_k^2 = a_k^2 + b_k^2 + c_k^2$$

$$\begin{bmatrix} \sum_k 1 - \frac{a_k^2}{m_k^2} & \sum_k - \frac{a_k b_k}{m_k^2} & \sum_k - \frac{a_k c_k}{m_k^2} \\ \sum_k - \frac{a_k b_k}{m_k^2} & \sum_k 1 - \frac{b_k^2}{m_k^2} & \sum_k - \frac{b_k c_k}{m_k^2} \\ \sum_k - \frac{a_k c_k}{m_k^2} & \sum_k - \frac{b_k c_k}{m_k^2} & \sum_k 1 - \frac{c_k^2}{m_k^2} \end{bmatrix} * \begin{bmatrix} x_c \\ y_c \\ z_c \end{bmatrix} = \begin{bmatrix} \sum_k x_k \left(1 - \frac{a_k^2}{m_k^2}\right) - \left(\frac{a_k b_k y_k}{m_k^2}\right) - \left(\frac{a_k c_k z_k}{m_k^2}\right) \\ \sum_k y_k \left(1 - \frac{b_k^2}{m_k^2}\right) - \left(\frac{a_k b_k x_k}{m_k^2}\right) - \left(\frac{b_k c_k z_k}{m_k^2}\right) \\ \sum_k z_k \left(1 - \frac{c_k^2}{m_k^2}\right) - \left(\frac{a_k c_k x_k}{m_k^2}\right) - \left(\frac{b_k c_k y_k}{m_k^2}\right) \end{bmatrix}$$

Interestingly, the pattern for extension to the n-dimensional case becomes again obvious when comparing it to the 2D case:

$$m_k^2 = a_k^2 + b_k^2$$

$$\begin{bmatrix} \sum_k 1 - \frac{a_k^2}{m_k^2} & \sum_k - \frac{a_k b_k}{m_k^2} \\ \sum_k - \frac{a_k b_k}{m_k^2} & \sum_k 1 - \frac{b_k^2}{m_k^2} \end{bmatrix} * \begin{bmatrix} x_c \\ y_c \end{bmatrix} = \begin{bmatrix} \sum_k x_k \left(1 - \frac{a_k^2}{m_k^2}\right) - \left(\frac{a_k b_k y_k}{m_k^2}\right) \\ \sum_k y_k \left(1 - \frac{b_k^2}{m_k^2}\right) - \left(\frac{a_k b_k x_k}{m_k^2}\right) \end{bmatrix}$$

These systems of linear equations can be solved by inverting $\varDelta$:

$$p_c = \varDelta^{-1} * \varTheta$$

For the 2D and 3D cases, we invert $\varDelta$ using closed-form solutions. Inverting higher-dimensional matrices can be achieved by computing the pseudo-inverse using Singular Value Decomposition[2].

2. Derivation for supporting axis-aligned ellipsoid (non-radial) objects

Radial Symmetry is, as the name suggests, designed for round objects where all gradients intersect exactly at the center point. However, it is desirable to support ellipsoid objects to support, for example, anisotropic datasets common in microscopy.

In microscopy, the pixel size in lateral (xy) and axial dimension (z) are not identical. The xy dimension is typically acquired with equal pixel size in x and y, using a camera or scanning device collecting light from an objective, while the step size in z is usually defined by a motor that moves the sample or the objective. Typically, the pixel size in z is bigger by a factor of 2-10. At the same time, the point spread function (PSF) of a microscopic system is also much smaller in xy compared to z. Although these two effects kind of balance each other, imaged single-molecule spots do not appear as round but ellipsoid objects in 3D.

We therefore derived a version of radial symmetry that supports axis-aligned, ellipsoid objects. We define the scaling for each gradient location as

$$\begin{pmatrix} x_k \\ u_k \\ v_k \end{pmatrix}^T \circ \begin{pmatrix} 1 \\ \alpha \\ \beta \end{pmatrix}^T = \begin{pmatrix} x_k \\ y_k \\ z_k \end{pmatrix}^T$$

where $q_k = (x_k, u_k, v_k)$ describes anisotropic coordinates in the input image, $s = (1, \alpha, \beta)$ describes the global known scale factors (anisotropy factors), $p_k = (x_k, y_k, z_k)$ describes the isotropic space coordinates in which radial symmetry will be computed and $\circ$ denotes element-wise multiplication (or Hadamard product). Mapping the gradients extracted from input images $\nabla I(q_k)$ at coordinates q_k into isotropic space with coordinates p_k yields:

$$\nabla I(p) = \begin{bmatrix} \frac{\partial I}{\partial x}(p) \\ \frac{\partial I}{\partial y}(p) \\ \frac{\partial I}{\partial z}(p) \end{bmatrix} = \begin{bmatrix} \frac{\partial I}{\partial x}(q) \\ \frac{\partial I}{\partial u}\frac{\partial u}{\partial y}(q) \\ \frac{\partial I}{\partial v}\frac{\partial v}{\partial z}(q) \end{bmatrix} = \begin{bmatrix} 1 \\ 1/\alpha \\ 1/\beta \end{bmatrix} \circ \begin{bmatrix} \frac{\partial I}{\partial x}(q) \\ \frac{\partial I}{\partial u}(q) \\ \frac{\partial I}{\partial v}(q) \end{bmatrix} = \begin{bmatrix} 1 \\ 1/\alpha \\ 1/\beta \end{bmatrix} \circ \nabla I(q)$$

In summary, the anisotropic coordinates q_k need to be scaled with the global scale vector s, while gradients extracted from the anisotropic input image $\nabla I(q_k)$ need to be scaled with the inverse of the global scale in order to compute radial symmetry on anisotropic datasets. Again, scaling to n-dimensional datasets is straightforward. Importantly, the resulting center point $p_c = (x_c, y_c, z_c)$ will be in isotropic space and might need to be scaled back into the anisotropic space.

3. Computing the global scale factor

For single-molecule detections, the global scale vector depends mainly on the pixel size and the PSF. For practical reasons, we only support the computation of the z scale factor β (called anisotropy factor) in the Fiji plugin. While assuming a scale of $\beta = 1.0$ might yield reasonable results since objects are usually roughly spheres, we highly recommend computing it for a specific set of microscope settings that are usually held constant across many experiments (same objective, same z stepping, same camera, same magnification).

The RS-FISH plugin offers two possibilities for computing the anisotropy factor. First, points can be interactively identified using Difference-of-Gaussian and a 3D Gaussian is fitted to each detection, which directly computes the anisotropy. Second, RS-FISH can sequentially run RS-RANSAC on a range of anisotropy factors (typically $0.1 - 5.0$). The correct anisotropy factor will be the one that maximizes the number of pixels used for spot detection since this indicates that most gradients intersect in the center of each spot. Both ways usually yield comparable results, which can be easily confirmed by manually inspecting single spots in 3D.

4. Accuracy benchmarking RS-FISH against commonly used tools

We compared RS-FISH's accuracy with the five most commonly used spot detection tools - FISH-quant (v3)[3], FISH-quant's python version - Big-FISH (0.5.0)[4], AIRLOCALIZE (1.6)[5], Starfish (0.2.2)[6], and deepBlink (0.1.1)[7] (**Fig. 2, Supplementary Table 1, 4**). The accuracy of spot detection was benchmarked using 50 simulated images, each of size 256x256x32 and containing either 30 or 300 simulated spots (**Fig. SN4.1a)**. For each tool, excluding deepBlink, we ran a grid search of the parameter space within a range of sensible values per parameter, running all value combinations between parameters. Parameter grid search was not run on deepBlink as it is an artificial neural network-based tool, however, we did run multiple configurations in the search for optimal results. Some of the benchmarked tools offer more than one spot detection pipeline. In such cases, we chose only one pipeline by following their documentation. Notably, this raises the possibility that superior accuracy and execution time were overlooked. This is especially relevant for Starfish, as Starfish is a tool for building image processing pipelines. The next section will detail the methods and grid search parameter value combinations (inclusive) for each tool.

All benchmarking scripts for all tools and instructions on how to reproduce are found here:
https://github.com/PreibischLab/RS-FISH/tree/master/documents/tool_comparison_for_paper
Instructions for installation and running the grid search are found in the README file in the link above.

RS-FISH
Pipeline: RANSAC
grid search parameters:

- DoGSigma = 1-2.5, step size += 0.25
- DoGthreshold = 0.001-0.13, step size *= 1.5
- supportRadius: 2-4, step size +=1
- inlierRatio: 0.0-0.3, step size += 0.1
- maxError: 0.1-2.6 step size += 0.5
- intensityThreshold: [0,100,150,200]

FISH-quant
Pipeline: Gaussian Fit
As FISH-quant is a MATLAB GUI application, optimal parameters were found manually for each image.
Parameter files and results can be found here:
https://github.com/PreibischLab/RS-FISH/tree/master/documents/tool_comparison_for_paper/FQ

Big-FISH
Pipeline: Dense region decomposition
As Big-FISH calculates a default value for each parameter automatically, the grid search range for some parameters was derived from the default value.
grid search parameters:

- sigmaZ: default + [-0.5,-0.25,0,0.25,0.5]
- sigmaYX: default + [-0.5,-0.25,0,0.25,0.5]
- threshold: value of index in threshold array, with indices relative to the location of the default. Relative to default indices: [-6,-3,-2,-1,0,1,2,3,6]
- alpha: 0.5-0.8, step size += 0.1
- beta: 0.8-1.2, step size += 0.1
- Gamma: 4-6, step size += 0.5

AIRLOCALIZE
Pipeline: 3DMaskFull

grid search parameters:

- Threshold = 2-13, step size += 1

minDistBetweenSpots = 1-3, step size += 1, additionally 0.2

Starfish

Pipeline: BlobDetector

grid search parameters:

Sigma: 2-5, step-size += 1

Threshold: 0.00004-0.000095, step size += 0.000001, and additional customized threshold values for images with insufficient accuracy.

deepBlink

Pipeline: deepBlink's pre-trained network "Particle"

DeepBlink is an artificial neural network-based tool for spot detection in 2D images, while all of our comparisons were performed on 3D images. However, since they offer a 3D adaptation solution and as it is currently the only notable tool based on deep learning, we decided to include it in the benchmarking analysis.

We have run multiple networks and found "particle.h5" to perform best on our simulated data (as well as the embryo images used in the execution time benchmarking detailed in the next section). The network outperformed deepBlink's pre-trained network "smfish.h5". It also outperformed networks that we trained from scratch, one specific network for each simulated image, using all available simulated images of the same signal-to-noise ratio as a training set. We suspect that the small training set and the small pool of spots were insufficient for the training task.

Analyzing grid search results

A grid search approach was used to find well-performing parameter combinations of RS-FISH and the other tools across 50 ground-truth simulated images, 38 images containing 30 spots each and 12-images containing 300 spots each. The dataset consists of images with a varying noise levels.

The resulting list of spot detections of each parameter combination of each software was evaluated by comparing it to a list of ground truth points. Both lists are 3D coordinates (x,y,z), with the size of the list being the number of spots. As FISH-quant returned spot location in nanometer instead of pixel coordinates, we converted the locations to pixel coordinates before running the comparison to ground truth. If necessary per tool, we first subtracted an offset from each detected spot coordinate to compare to ground truth spot coordinates. We found the correct offset for each tool by centering the delta of the distance in each dimension (X, Y, Z) separately around zero (**Fig. SN4.2d-e**). The two tools that required offsets were AIRLOCALIZE and Big-Fish - (-0.5,-0.5,-1) and (-0.25,-0.25,-0.25), respectively. Importantly, ground truth spot coordinates from LOC files were offset by (-0.5,-0.5,-1) for all tools (hence AIRLOCALIZE outputted spot locations and original ground truth coordinates are the same.

For the comparison of detected spots with the ground truth, a KD-Tree of all detected points was constructed. All ground truth points were queried against the KD-Tree. The ground truth list element with the smallest distance between itself and a detection was deleted from the ground truth list. The corresponding detection element was also deleted from the detection list. This process was repeated until there were either no elements left in the ground truth list, no elements left in the detection list, or the smallest distance between a ground truth point and elements in the detection array was above a certain threshold (a distance of *2.0* pixels was used).

Once an end condition was met, the performance of the parameter combination was assessed (**Supplementary Table 1**). The performance was measured using the length of the ground truth list after measurement (how many ground truth points have not been detected), and the length of the detection list after measurement (how many false detections have been made), and the average Euclidean distance between the detected spots and their associated ground truth (true positives only).

The best performance of each software for each image was selected by choosing the parameter combination results that maximized the F1 score:

$$F1\ Score = \frac{2\ x\ Precision\ x\ Recall}{Precision\ +\ recall}$$

Where:

$$Precision\ = \frac{TP}{TP + FP}$$

$$Recall\ = \frac{TP}{TP\ +\ FN}$$

Thus:

$$Fi\ score\ = \frac{TP}{TP\ +\ 0.5\ (FP\ +\ FN)}$$

In case multiple parameter combinations resulted in the same performance using the F1 score metric, the average Euclidean distance between detections and their associated ground truth point was also taken into account, with the best results having the lowest mean Euclidean distance. We compared the performance for each tool across ground truth simulated images containing different numbers of points and varying background noise (**Supplementary Table 1**).

Supplementary Table 1 (data in XLS file): Comparison of localization performance on simulated data per image. The metrics used are the F1 score and the mean Euclidean distance (in pixels) between the ground truth location to each detected spot. The tools compared are AIRLOCALIZE (AL), Big-FISH (BF), deepBlink (DB), FISH-Quant (FQ), RS-FISH (RS), and Starfish (SF). RS-FISH assumes that the intensity measurements of a pixel are located at for example 0.0, 0.0, 0.0 (the first pixel), and outputs all locations in pixel values.

The data is available as external Excel file (XLS) available for download with the manuscript.

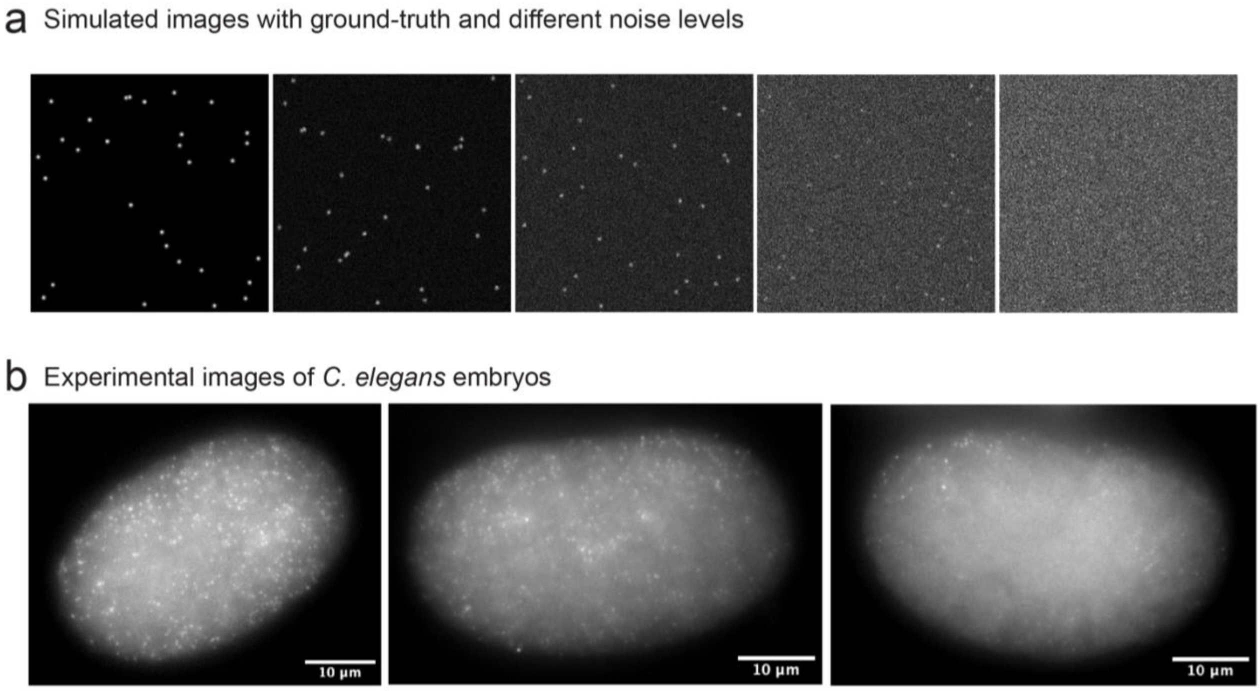

Figure SN4.1: *Example images used for benchmarking.* ***(a)*** *Examples of simulated images (size 256x256x32) with either 30 or 300 points with different noise levels. The stdev of image noise ranges from 0 (left image) to 8 (right image). Points are located randomly in 3D, and each spot's brightest pixel intensity was picked from a normal distribution. Full dataset includes 50 images* ***(b)*** *Examples of experimental images of C. elegans embryos at different developmental stages. The embryos are stained with smFISH for different genes expressed throughout embryogenesis. Different probes were labeled with fluorophores, including Quasar 670, CAL Fluor Red 610, or Quasar 570. Full dataset includes 13 images from 3 biological replicates.*

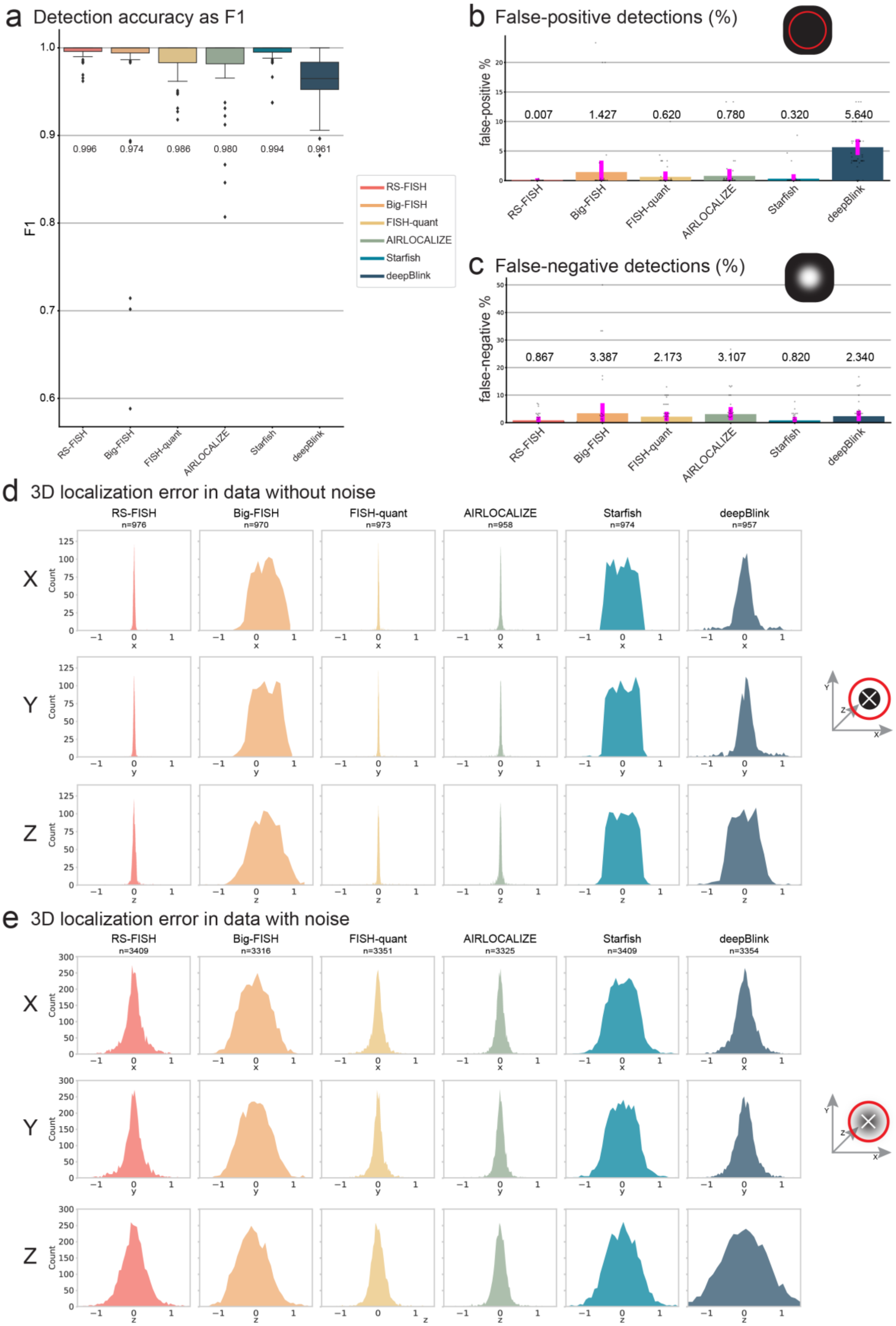

Figure SN4.2: *Benchmarking with simulated images.* ***(a)*** *The accuracy of detection for different tools measured in the F1 score corresponding to Fig. 2 shown as boxplots with the full range of outliers. The center of the box plot depicts the mean, the box size the quartiles of the dataset, the whisker show the rest of the distribution excluding outliers which are shown as single points.* ***(b)*** *False-positive (FP) detection percentage for wrongly detected spots using simulated data.* ***(c)*** *False-negative (FN) detection percentage for missed detections using simulated data. The bar plots in b and c show mean (also written as number) and a 95% confidence interval (pink bar) of the 50 measured detections* ***(d)*** *Histograms depict the delta of the distance of ground truth to the detected spot* $(GT - detected)$ *for each dimension. 6 images without noise (images with StDev of image noise = 0) were used for this analysis.* ***(e)*** *For each image dimension, a histogram of the delta of the distance of ground truth to the detected spot of all spots in the analysis for images with noise (images with StDev of image noise of 1-8). In d and e, the number of localizations (n) is depicted above the plots.*

5. Computation time benchmarking

To compare computation time between RS-FISH, FISH-quant (v3)[3], Big-FISH (0.5.0)[4], AIRLOCALIZE (1.6)[5], Starfish (0.2.2)[6], and deepBlink (0.1.1)[7], we performed several realistic smFISH localization runs on 13 3D images of *C. elegans* embryos (**Supplementary Table 2, Fig. SN4.1b**). Image size varied between images with lateral size (XY) of 334-539 pixels and axial size (Z) of 81-101 pixels. Since execution times significantly depend on the number of detected spots, we used settings that produce a comparable number of spots in all tools, excluding deepBlink. deepBlink spot detection was performed using its pre-trained artificial neural network “Particle”, thus, the approximate number of spots found was fixed. Notably, the difference in the results to other methods may be attributed to a mismatch between the chosen pre-trained network and the spot detection task on the embryo images, however, we did not have training data to train the network from scratch. Additionally, as deepBlink is based on networks to analyze 2D images, a sizable portion of each computation time that is reported was exhausted by the step that adapts the network’s results from 2D to 3D, and while this step is offered as part of deepBlink’s package, it is not part of the main pipeline.
In the metric of computation time, we found that RS-FISH significantly outperforms all compared tools.

Link to download embryo images:
https://doi.org/10.6084/m9.figshare.21067369.v1

The computation time benchmarks for all tools were run on a Dell Precision Tower 7910 workstation, Intel(R) Xeon(R) CPU E5-2640 v4 @ 2.40GHz. deepBlink spot detection was run with TensorFlow GPU on Nvidia Quadro P6000.

Supplementary Table 2 (data in XLS file): *Comparison of execution time of real 3D datasets. The tool’s parameters were set for each image so that the number of detected spots was comparable across tools (excluding deepBlink, as no parameters could be set). The tools compared are AIRLOCALIZE (Air), Big-FISH (BF), deepBlink (DB), FISH-quant (FQ), RS-FISH (RS), and Starfish (SF).*

The data is available as external Excel file (XLS) available for download with the manuscript.

Supplementary Table 3: Information on datasets used for this study.

dataset	organism	short description	reference
Fig 1c	*C. elegans*	C. elegans larvae labeled with smFISH for LEA-1 mRNA. https://doi.org/10.6084/m9.figshare.21067342.v1	This study
Fig 1d	mouse (mESC)	Mouse embryonic stem cell labeled with DAPI (blue) and smFISH for *Cdx-2* mRNA (white). https://doi.org/10.6084/m9.figshare.21067360.v1	This study
Fig 1e	*Drosophila*	*Drosophila* brain labeled with smFISH for *Pura* mRNA. https://doi.org/10.6084/m9.figshare.21067366	This study
Fig 1f	human (PGP1f cells)	Images correspond to four channels of a single round of OligoFISSEQ labeling with the 36plex-5K oligopaint library (Fig. 2 in Nguyen et al.[9])	Nguyen et al.[8]
Fig 1g	*C. elegans*	One example image of a *C. elegans* embryo labeled with smFISH for MDH-1 mRNA of a dataset with 4010 mixed stage embryos. https://doi.org/10.6084/m9.figshare.21067354	This study
Fig 1h, Supplementary Video 1	mouse	Expansion-Assisted Iterative Fluorescence In Situ Hybridization (EASI-FISH) spots for *Map1b* mRNA detected in a tissue section of the lateral hypothalamic area.	Wang et al.[9]
Fig 2 a-c, e,f; Fig. SN4.1a, SN4.2; Supplementary Table 1	simulated	Simulated data of diffraction-limited spots with varying degrees of background noise. https://github.com/PreibischLab/RS-FISH/tree/master/documents/Simulation_of_data	This study
Fig 2d, Supplementary Table 2	*C. elegans*	13 *C. elegans* embryos at different developmental stages labeled with smFISH for different mRNAs. https://doi.org/10.6084/m9.figshare.21067369.v1	This study
Fig. SN 7.1,2	simulated	Simulated data of close diffraction-limited spots with varying degrees of background noise. https://github.com/timotheelionnet/simulated_spots_rsFISH/tree/main/out	This study
Fig.SN6.1	human (Kuramochi cells)	Kuramochi cells stained for *IGF2* mRNA imaged with different acquisition times resulting in noise levels https://bimsbstatic.mdc-berlin.de/akalin/preibisch/FISH_real_w_noise_levels.zip	This study

Supplementary Table 4 (data in XLS file): Comparison of localization performance on simulated data per image. The metrics used are the F1 score - calculated using the number of spots in the image (n_spots), the number false negative detections (FN), and the number of false positive detections (FP) - and the mean Euclidean distance (in pixels) between the ground truth location to each detected spot. The tools compared are AIRLOCALIZE (AL), Big-FISH (BF), deepBlink (DB), FISH-Quant (FQ), RS-FISH (RS), and Starfish (SF).

The data is available as external Excel file (XLS) available for download with the manuscript.

Supplementary Table 5 (data in XLS file): Comparison of localization performance on real smFISH data per image and channel. The metrics used are the F1 score - calculated using the number false negative detections (FN), the number of false positive detections (FP), the number of consensus spots across tools on the best SNR image as ground truth (n_spots_c3conse), and the number of found spots (n_spots) - and the mean Euclidean distance (in pixels) between the ground truth location to each detected spot. The tools compared are AIRLOCALIZE (AL), Big-FISH (BF), FISH-Quant (FQ), RS-FISH (RS), and Starfish (SF).

The data is available as external Excel file (XLS) available for download with the manuscript.

6. Benchmarking on real data

To determine the influence of noise in real smFISH images on the detection accuracy, we imaged Kuramochi[10] cells labeled with smFISH in the same field of view with decreasing image acquisition times leading to different SNR. In total, 63 2D images of size 512x512 pixels (81.92x81.92 µm) were used for the analysis. Each image was imaged with four noise levels and a DAPI channel (the DAPI channel was not used in the analysis) (**Fig. SN6.1a)**. The highest noise level was excluded from the analysis as it was too noisy to detect most spots, thus three noise levels were used. Since real data does not have ground-truth information on the true count and localization of points, we reasoned that the points in the best SNR image found by all spot detection tools would give us a good set of points to detect missing spots. Using this SNR level as "ground-truth" information, we manually found the best parameters for each tool for each noise level on one example image and used those parameters for all images of that noise level. With this approach, we could only report false-negative detections since we could not assess if extra points were true spots or false-positive detections. While we tried to find the best parameters for each tool, we admit that this analysis was sensitive to chosen parameters. Additionally, optimizing the detections to have as few false-negative detections as possible can skew the detection into over detection. We excluded deepBlink from the analysis as it performed substantially worse in our first benchmarking approaches (**Fig. 2**) and did not detect most spots that all other tools detected in the current analysis. Nevertheless, RS-FISH can detect spots well also in real data with low SNR (**Fig. SN6.1b, Supplementary Table 5)**.

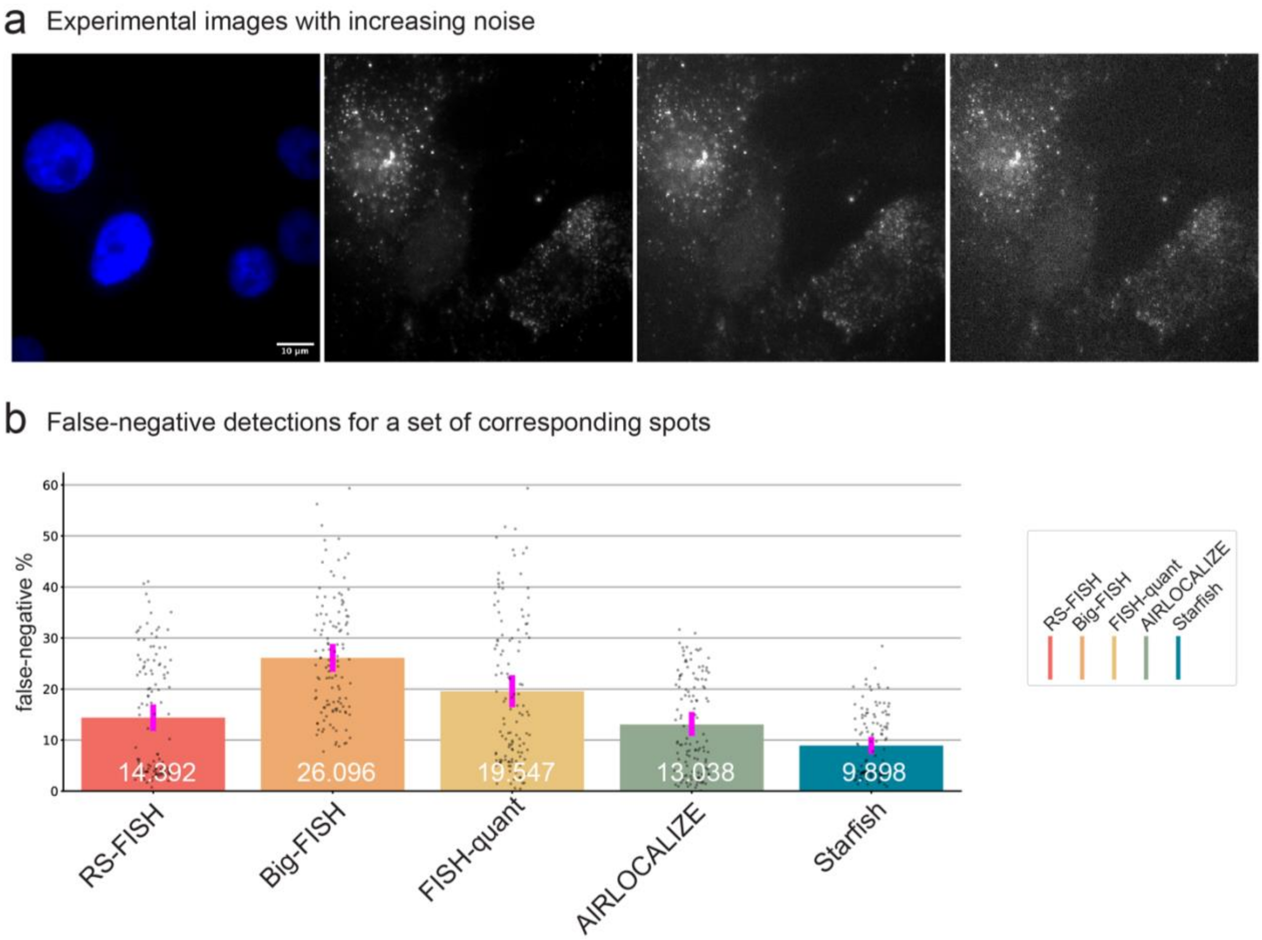

Figure SN6.1: *Benchmark with real images* ***(a)*** *Experimental smFISH images of Kuramochi cells staining with DAPI (first image) and smFISH probes against IGF2 mRNA. The same field of view was imaged multiple times (images 2-4) with different acquisition times leading to different noise levels. Full dataset includes 63 images.* ***(b)*** *False-negative detection percentage for missed detections using real data as described above. The lowest noise level images (best SNR) were used to detect points using all tools, and the minimum set of points found across all tools was used as a ground truth. False-negative detections were analyzed per image per tool using the two higher noise level images for all images (n=63) in the dataset. The bar plots show mean (also written as number) and a 95% confidence interval (pink bar).*

7. Close spots analysis

To test the performance of RS-FISH for very close points, we simulated a set of images (n=720) with 30 diffraction-limited points in each. As shown in **Figure SN7.1a**, the points were arranged as 15 pairs, with different distances between the paired spots (from 0.25 - 4 pixels), and the analysis included images with different SNR. Images were generated similarly to the data simulation described in this article's main text. Thus, each of the 15 pairs of close points position was randomly located in each image in the dataset. All images were of 256x256x32 pixels. As image and spot properties were similar to the previously described simulated dataset, we performed the same grid search over the same parameter space as described in section 4 above for all tools. RS-FISH includes a multi-consensus RANSAC feature that improves performance in dense datasets by detecting multiple points within each local patch (see also "Choosing the right parameters for RS-FISH"). Thus, we enabled multi-consensus RANSAC and added its parameters to the RS-FISH grid search. However, in an effort to limit the parameter space for RS-FISH, we only ran the multi-consensus RANSAC grid-search in combination with parameter combinations (listed in section 4) that performed best on at least one image. Comparison of the resulting detections to ground-truth for each tool for each image was performed as described in section 4. The results of this analysis proclaim that RS-FISH is superior in detecting close spots, calculated by the F1 metric (**Figure SN7.1b, Supplementary Table 6)**. A detailed performance analysis per distance of close spots per noise level can be found in **Figure SN7.2**. RS-FISH performs worst on the average Euclidean distance to ground truth spots, but this can partly be explained by RS-FISH detecting more spots than any other tool in this dataset (**Figure SN7.1c)**.

RS-FISH (additional parameters)
Pipeline: Multi-consensus RANSAC
grid search multi-consensus RANSAC parameters:

minimum number of inliers = 20
initial inliers = [4,5,6,7,8,9,10,11,12,13,14,15,16,17,18,19,20]
final inliers = [3,3,4,5,6,6,7, 8, 9, 9, 10,11,12,13,14,15,16]
Importantly, the combination of initial and final inliers tested in the grid-search are not crossed between the two lists above. Instead, only pairs of the same index from the two lists were used.

Supplementary Table 6 (data in XLS file): Comparison of localization performance on simulated close spots data per image. The metrics used are the F1 score - calculated using the number of ground truth spots (n_spots), false negative detections (FN), and false positive detections (FP)- and the Euclidian distance (euc_dist) of the detection to the ground truth spots. The tools compared are AIRLOCALIZE (AL), Big-FISH (BF), FISH-Quant (FQ), RS-FISH (RS), deepBlink (DB) and Starfish (SF). The images had a standard deviation of image noise (std_noise) from 1-4 and a distance between the spot pairs (adj_dist) from 0.25 – 4 pixels.

The data is available as external Excel file (XLS) available for download with the manuscript.

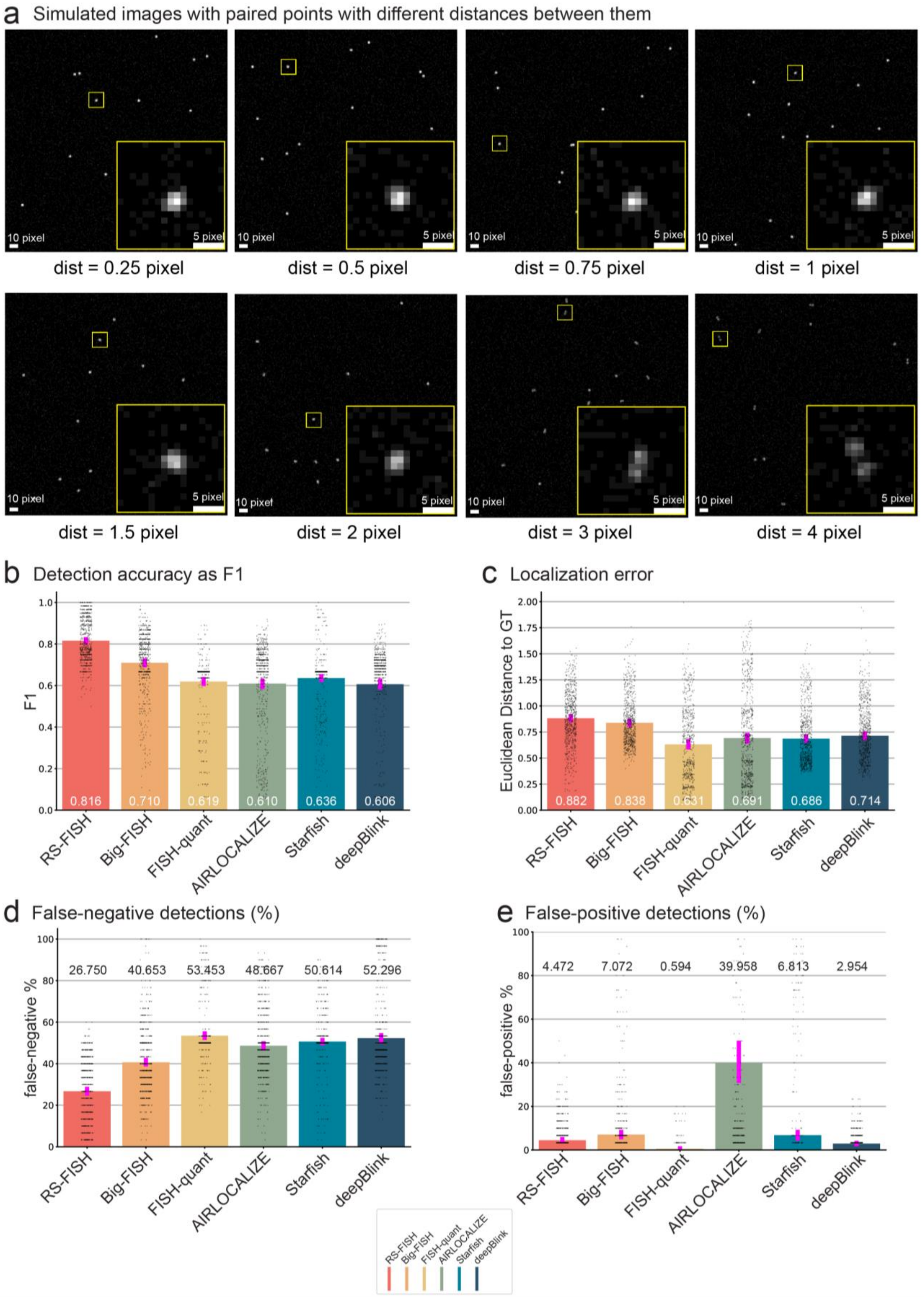

Figure SN7.1: *Detecting close spots.* ***(a)*** *Max projections of simulated 3D example images of diffraction-limited spot pairs, each with different 3D distances (dist) between the pair. Each image contains 15 pairs of spots (a total of 30 spots). The box in the lower right part of the images shows a magnification of one point pair for each image.* ***(b)*** *Detection accuracy is measured as the F1 score, calculated from true-positive, false-positive (in e), and false-negative detection (in d).* ***(c)*** *Localization error is measured as the Euclidean distance between the detected center and the ground-truth point center.* ***(d)*** *Percentage of false-negative detections.* ***(e)*** *Percentage of false-positive detections. Images (n=720) used for the analysis in panels b – e had 15 spot pairs with distances shown in part a and different SNR. The bar plots in b - e show mean (also written as number) and a 95% confidence interval (pink bar).*

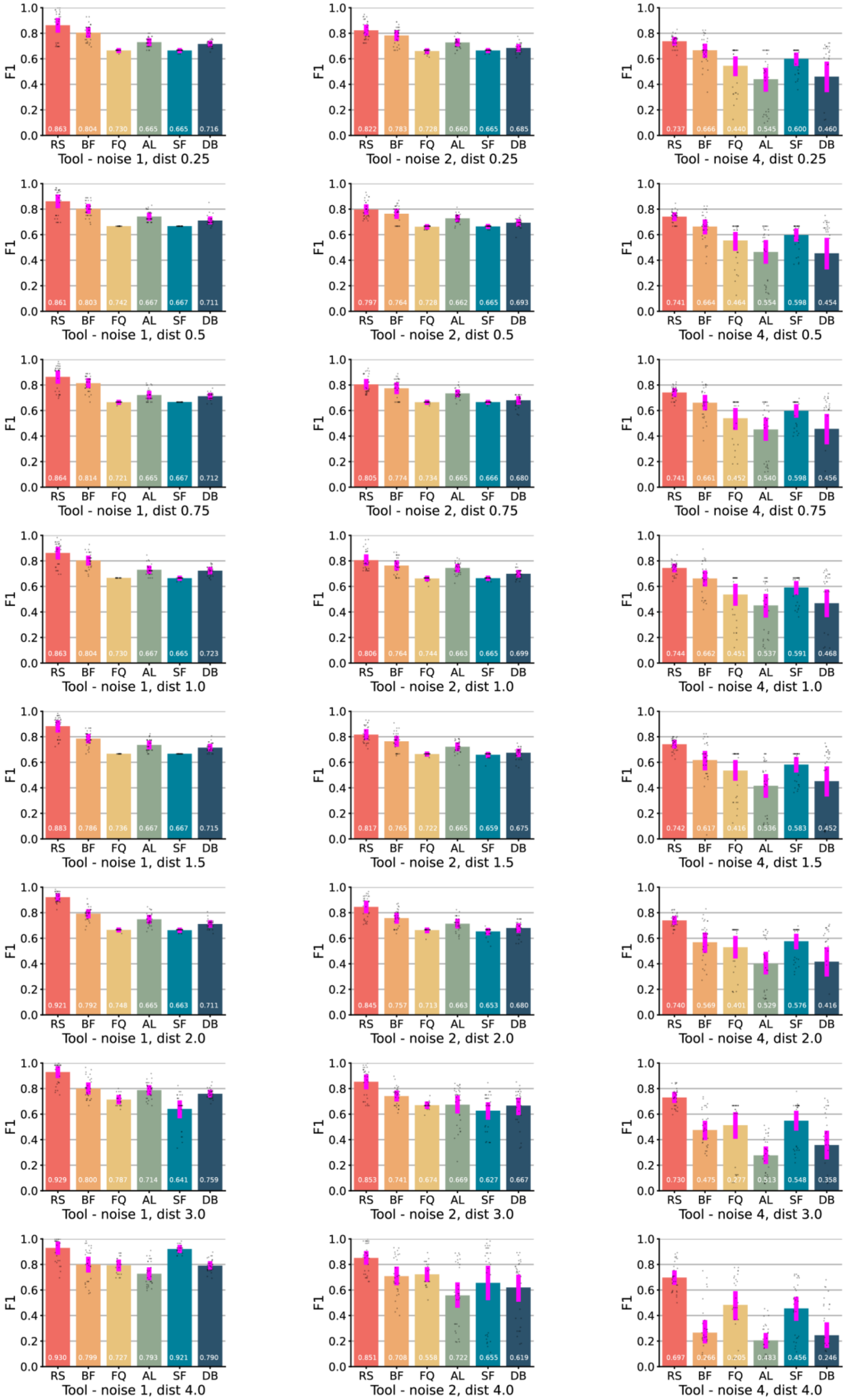

Figure SN7.2: *Extended detection accuracy result plots from Fig. SN7.1.. Each row of results plots displays different distances (dist) between pairs of points ranging from 0.25 to 4. Each column of results plots displays different image noise levels from 1 to 4 (corresponding to the std of image noise in Fig. SN4.1). The tools compared are RS-FISH (RS), Big-FISH (BF), AIRLOCALIZE (AL), FISH-Quant (FQ), Starfish (SF) and deepBlink (DB). The bar plots show mean (also written as number) and a 95% confidence interval (pink bar). For each combination of distance and image noise, 30 images were used in the analysis.*

8. Choosing the right parameters for RS-FISH

RS-FISH allows the user to choose between different detection methods to localize spots in the image. The tool offers three localization methods that build on each other: 1. radial symmetry ('No RANSAC'), 2. radial symmetry + RANSAC (the default option), 3. radial symmetry + multi-consensus RANSAC (**Fig. SN8.1a**). The default option is radial symmetry detection of spots with additional RANSAC outlier removal. In cases where the noise level is low, and an even higher computation speed is preferred, the RANSAC outlier removal step can be skipped by choosing "no RANSAC". In cases where many spots are crowded and potentially overlap, we recommend choosing multi-consensus RANSAC spot detection, which will try to separate close points by detecting gradients in several rounds.

The next step is to choose the parameters for detection, which is simplified by the plugin's visual feedback (**Fig. SN8.1b**). Using the default spot detection method, there are two windows with parameters to set and two different visual feedback cues (see the **Tutorial RS-FISH** section). The red circle marks spots detected using the radial symmetry parameters, and the blue cross confirms those spots that pass the RANSAC outlier removal. The detection of the red circle can be adjusted by tuning the expected dimensions of the spot (*sigma*) and the *threshold* of the signal intensity of the DoG pre-detected spots.

The RANSAC parameters determine which and how the gradients are used to localize each spot. The *support region radius* determines the number of gradients computed in a local patch around each DoG pre-detected spot. We propose a default radius of 3 pixels which means a 7x7[x7] pixel patch resulting in 216 gradients for the 3D case. In general, we propose to select this radius to have a size similar to the radius that was selected for DoG (red circle). Decreasing this number might allow more spots to be found but will decrease localization precision as fewer gradients are used to define the center. The *inlier ratio* describes the fraction of gradients computed in each patch that are required to converge to the same center point in order to retain the pre-detected spot as a valid localization. Increasing the *inlier ratio* increases the robustness of the detected spot but can decrease the total number of detected spots, especially for lower SNR spots. The *max error* threshold defines the maximally allowed distance error (in pixels) for the intersection point defined by the gradients in order to classify a given gradient as an inlier; i.e., how far away from the intersection point can each gradient be (only in a perfect scenario, all gradients intersect perfectly in the same point, see **Fig SN1.1d**). Decreasing the *max error* decreases the number of false detections (because gradients generated by a noisy background rarely point towards the exact same center), but potentially also reduces the number of lower intensity real detections, especially if the noise level is high. The *max error* should be chosen as function of noise, the lower the noise, the lower the error can be. We generally suggest an error around 1 pixel as a good starting point.

For the multi-consensus selection, a window pops up where parameters for additional rounds of spot detection can be defined. It is important to realize that for multi-consensus to work well, the *max error* in the RANSAC settings should be set relatively low since otherwise, the gradients pointing towards the centers of two (or more) nearby spots are interpreted as intersecting into a single spot. A typical setting could be *0.5 - 0.75 pixels* for images with good signal-to-noise levels. It is important to first run RS-FISH in RANSAC mode before running the multi-consensus because the output parameters of the first RANSAC run are used to set the multi-consensus parameters. Upon finishing the first RANSAC run, the log record will output two critical parameters: the *average #inliers* and *stdev #inliers*. The *average #inliers* defines how many inliers intersect in each spot on average, while the *stdev #inliers* describes its variability. The parameters for multi-consensus RANSAC must be defined relative to those two values. The *"Initial #inlier threshold for new spot (avg - n*stdev) n=*" asks the user how many inliers are required for it to be considered a new spot next to an already found one. For example, let us assume a 7x7x7 local neighborhood with 216 total candidate gradients. If *average #inliers* was 80 and *stdev #inliers* was 6, then defining *n=8* would require at least 80-48=32 inliers for a new spot to be defined. Keep in mind

that only around 136 gradients (=216-80) are left to be evaluated per local patch because about 80 are already assigned to the first spot. Once a new spot is identified, we re-center the local patch around its center location and compute the gradients across the updated local patch. Thus, the second parameter, "*Final #inlier threshold for new spot (avg - n*stdev) n=*", is usually chosen higher, e.g., *n=6*, thus requiring 80-32=48 inliers in the updated patch for a final confirmation of a new spot.

Setting the *min number of inliers* is a simpler, not adaptive way to define when new spots are assigned in a local patch. It describes the absolute number of inlier gradients minimally required to localize a point. Typically, a value of *30 inliers* is a reasonable starting point. Note that in order to only use the *min number of inliers*, it is required to set *"Initial #inlier threshold for new spot (avg - n*stdev) n=*" and "*Final #inlier threshold for new spot (avg - n*stdev) n=*" very high.

The entire process can be automated by starting *Plugins > Macro > Record* before running RS-FISH. Note that interactive dialogs are not recorded. However, values that were selected in interactive mode are remembered when running advanced mode afterwards. Thus, a typical workflow is to first set all parameters interactively and make sure the results are as desired. Subsequently, start macro recording and run RS-FISH in advanced mode, which will be fully recorded. After clicking "create", the macro editor appears that allows to save and run the macro. The macro can then be modified for example for batch processing. For a full explanation of macro capabilities please consult https://imagej.nih.gov/ij/developer/macro/macros.html.

For large data processing it is required to save the dataset in the N5 format. For images that fit into the RAM of the machine, you can simply open them and select *File > Save As … > Export N5*. For larger datasets you can for example use *BigStitcher* (https://imagej.net/plugins/bigstitcher/) to resave images as N5, or export stitched datasets as N5 using *BigStitcher-Spark* (https://github.com/PreibischLab/BigStitcher-Spark). If you want to write your own scripts you can use the *n5-utils* project as a starting point (https://github.com/saalfeldlab/n5-utils).

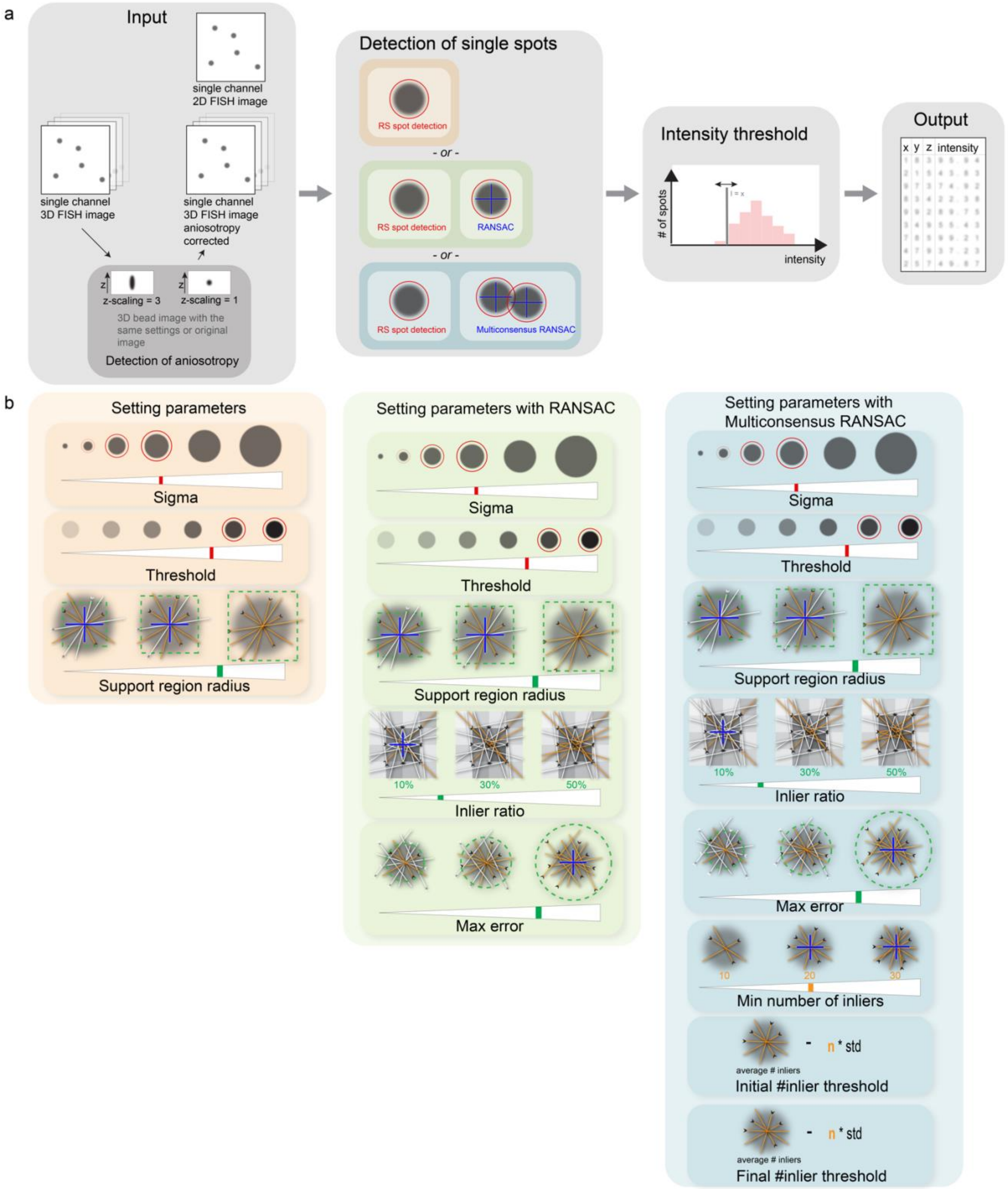

Figure SN8.1: *Workflow for RS-FISH.* ***(a)*** *RS-FISH has four major steps. Firstly, choose an input image that is either 2D or 3D. If a 3D image is chosen, the anisotropy can be detected using our anisotropy detection tool. This value is used to virtually correct the z-axis. Next, depending on the selected detection method, different parameters can be used to find all FISH spots (explained in more detail in b). For very fast detection, RS mode can be selected. For more precise detection, an additional outlier removal step using RANSAC can be selected. For an image with very dense spots, choosing Multiconsensus RANSAC can be helpful to separate close points. Next, a global intensity threshold can be used to filter out detections. In the last step, RS-FISH returns a Result table that can be saved for downstream analysis.* ***(b)*** *Depending on the selected detection method, different parameters can be chosen. The radial symmetry parameters sigma and threshold influence the red circle during the interactive parameter setting to select the right size and intensity of spots (dark circles). The blue cross marks spots selected by the current RANSAC parameters in the interactive GUI. The RANSAC parameters choose which and how the inlier gradients (orange) are used.*

9. Tutorial RS-FISH

Calculating Anisotropy Coefficient

Since the effective size of objects along the z-axis is usually different from the x- and y-axis of many images, it should be corrected to achieve a more accurate smFISH detection. To estimate your anisotropy coefficient, you can acquire a fluorescent bead image on the same microscope, using the same settings and equipment, or you can use the smFISH image directly.

Open the image with the beads or the smFISH detections and navigate to the 'Plugins' > 'RS-FISH' >'Tools'> 'Calculate Anisotropy Coefficient'.

You will see the dialog window in **Fig. SN9.1a**. Make sure your bead image is selected in the **Image** drop-down menu. Next, you can choose between two **Detection methods: Gauss fit** or **Radial Symmetry.** If you have fewer detections, Gaussian fit might be the better choice; however, both methods usually provide reasonable results. It can even be beneficial to simply average the results of both approaches. The resulting number can be visually confirmed by turning the input image around its x or y-axis (Image > Stacks > Reslice > Top) as it simply describes the ratio of the size in Z versus XY. After choosing a detection method, two windows will open once you press **OK**.

In the **Adjust difference-of-gaussian values** window (**Fig. SN9.1b**), you can choose **Sigma** and **Threshold** values to detect the majority of subpixel resolution spots.

Once you are done – press the **Done** button.

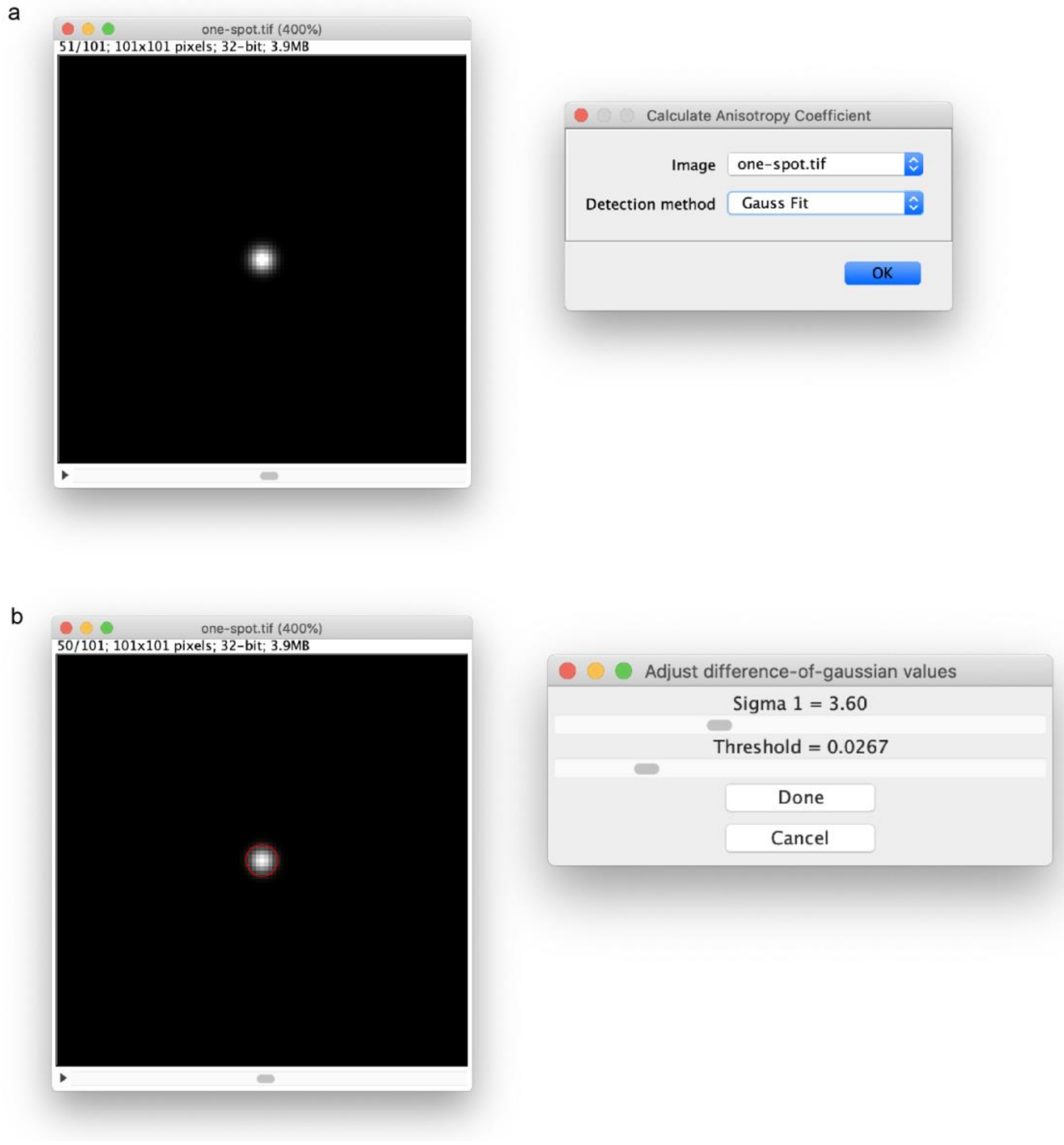

Figure SN9.1. *Calculating the Anisotropy Coefficient of a fluorescent image***.** *a) Selecting a bead or smFISH image for anisotropy coefficient detection. b) Adjusting the difference-of-gaussian values to detect single spots for anisotropy coefficient calculation.*

Depending on the number of spots, the calculations might take some time as the Gaussian fit is slower, and the RS-RANSAC needs to iterate over a range of potential anisotropy coefficients. The program will calculate the corresponding anisotropy coefficient, which shows how we should squeeze the objects in the z-axis to make them look radially symmetric.

The Log window will show the corresponding anisotropy value, and it should be automatically transferred to the next step.

Important: It is OK to skip this step if the objects are more or less round in 3D. The plugin will do a decent job even with the default value of the anisotropy coefficient. However, we advise performing this before the actual RS detection.

Localizing Spots

The main RS-FISH plugin can be found under Plugins > RS-FISH > RS-FISH. There are two different modes of processing images: **interactive** and **advanced**. The Interactive method is used to adjust the parameters for further dataset processing or is the right choice if single images need to be processed. The interactive mode provides the visual feedback necessary to adjust the advanced automated processing parameters on large datasets.

Interactive mode

Open a 2D or 3D single-channel image for analysis and navigate to the 'Plugins' menu under 'RS-FISH' > 'RS-FISH'. A window will pop up, as depicted in **Fig. SN9.2a**. Ensure that the correct image is chosen in the **Image** drop-down menu. Next, you choose the **mode** that you want to run RS-FISH in. For finding the best parameters or analyzing a small set of images, select the **Interactive Mode**.

Anisotropy coefficient defines how much the z-resolution differs from the x/y-resolution. The parameter would be set automatically if you ran the '**Calculate Anisotropy Coefficient**' plugin beforehand (Plugins > RS-FISH > Calculate Anisotropy Coefficient'). In general, 1.0 gives a good result if the spots are somewhat round in 3D. You can use the same anisotropy coefficient for computing the Difference of Gaussian (DoG), which will lead to a more robust DoG detection for anisotropic spots.

There are various options for **Robust fitting Computation.**

- '**RANSAC**' defines if you want to use radial symmetry with robust outlier removal, it will identify all gradients within every local patch that supports the same center point (**Fig. 1**)
- '**No RANSAC**' for the use of radial symmetry without robust outlier removal, simply all gradients of a local spot will be used to compute the center point (classic RS)
- '**Multiconsensus RANSAC**' will iteratively run robust outlier removal on each local patch until all sets of gradients are identified that support a center point. This allows RS-FISH to potentially find multiple points within each local patch identified using DoG detections.

The next option you can choose is to **compute the min and max intensity from the image**. If you deselect the option, in the next step, you can choose a range of intensity of the image, which might be a good option if you are processing a set of images with the same acquisition parameters.

There are two options in RS-FISH to calculate the spot intensity value. Each spot's associated intensity values are by default computed using linear interpolation at the spot's sub-pixel location. By selecting the option **Refine spot intensity with Gaussian fit on inliers,** the spot intensity can be refined by fitting a Gaussian to the subset of pixels that support the spot as identified by RS-RANSAC.

The last option during this first step is to visually select the spots from an Intensity histogram in the **Visualization** section. This option is only available in the interactive mode. This option will allow you to choose the found smFISH spot by thresholding based on an intensity value. Once you are done with the settings, press the **OK** button.

In the second step, multiple windows will open based on your selection (**Fig. SN9.2b**).

In the **Difference of Gaussian** window, you can adjust the parameters for the initial detection of the spots. The goal of this step is to minimize false detections. Adjust the **Sigma** and **Threshold** slider so that the red circles in the image detect as many single spots as possible. Try to slightly find more spots if you choose RANSAC; the RANSAC window allows additional restrictive settings. If you are working with a 3D stack, it helps to move through z while adjusting the parameters, as the red circle appears only in the z-slices where the signal is the strongest. It can help to change the yellow preview box during this step. If the image is very large, it can help to choose a smaller box to speed up the visualization (the detection will be performed in the whole image).

Important: If you choose to run RANSAC robust fitting, do not click the Done button on the Difference of Gaussian window at this step; simply continue setting the parameters in the Adjust RANSAC values window.

The **Adjust RANSAC values** dialog allows you to find the right setting for the robust outlier removal. The **Support Region Radius** defines the radius for each spot in which gradients are extracted for RS. You might want to play with this parameter. Sometimes it is helpful to increase the radius and decrease the **Inlier Ratio** simultaneously. The **Inlier ratio** defines the ratio of the gradients (i.e., pixels) that have to support the RS center point (Simply speaking, the ratio of pixels should 'belong' to the current spot), given the **Max error** that defines maximally allowed error for RS fitting (see **Fig. 1**).

While moving the sliders, you will see the updates in both image windows. Firstly the **RANSAC preview** window displays the pixels used by RANSAC and the error values at each of the used pixels. The second window is the initial image window with the preview of the detections. Additionally to the red circles, the blue crosses indicate spots detected using RANSAC outlier removal. So the goal of this part is to find all spots with a red circle and a blue cross inside while not detecting noise or background.

The background removal step allows you to remove a non-planar background prior to computing the RS. It will try to estimate a plane using the intensity values of the edges of each local patch using any of the outlined methods. *Note: constant backgrounds do not need to be removed, only if strong gradients are present.*

Once the parameters are adjusted, hit any of the 'Done' buttons and wait a bit while the computations are performed.

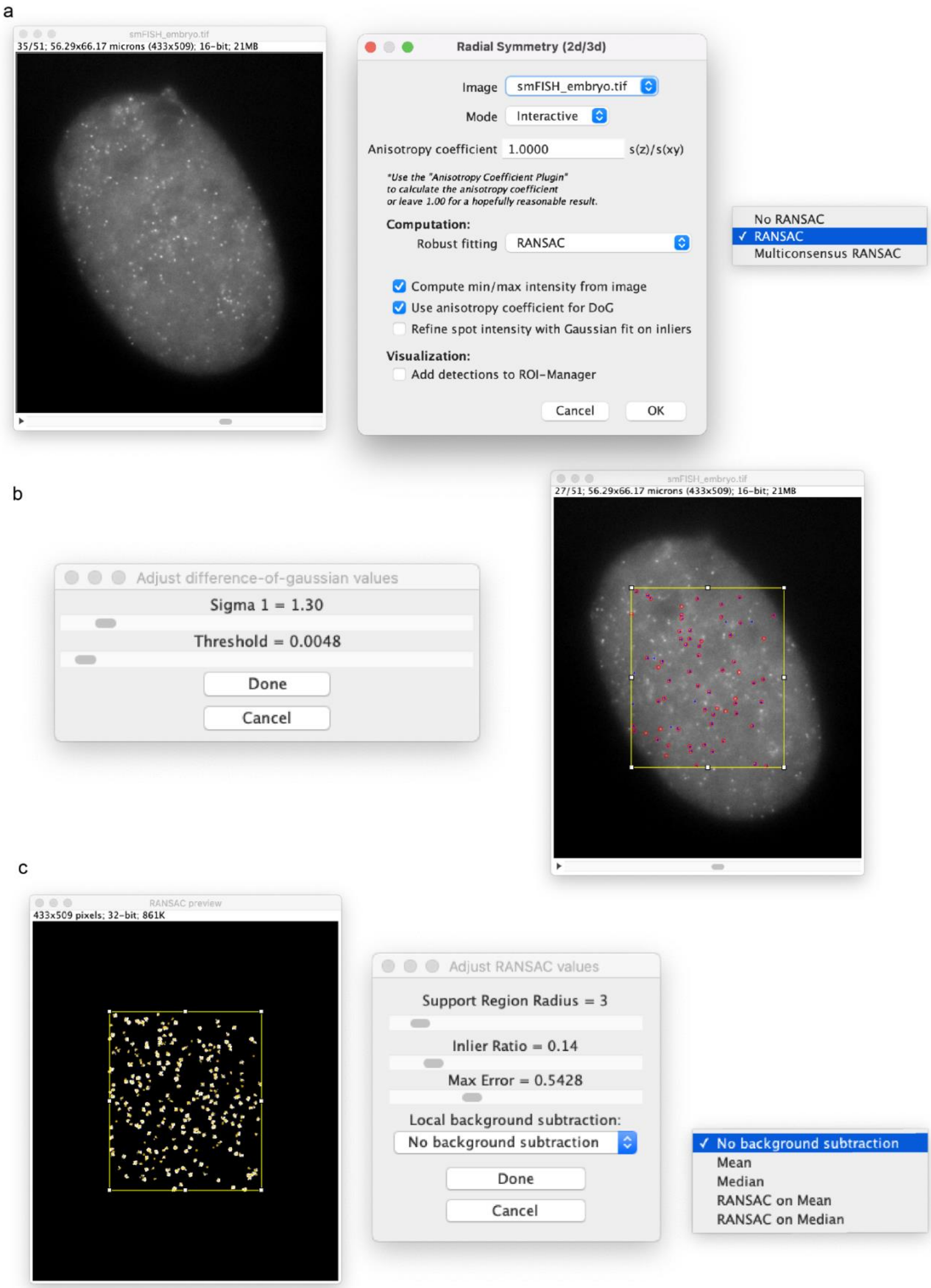

Figure SN9.2*: The RS-FISH interactive mode for spot detection***.** *a) With an open smFISH image, navigate to the RS-FISH plugin and choose the interactive mode. Choose the computation mode from the drop-down menu to fit the spots. b) Adjust the difference-of-gaussian values using the sliders to detect single spots in your selected image within* the yellow selection box. c) Adjust the RANSAC values for improved detection accuracy and correct an uneven background if necessary.

In the next window (**Fig. SN9.3a**), you can threshold the detected spots based on their intensity. The **Intensity distribution** window displays all detected spots and their corresponding intensity value as a histogram. The blue thresholding bar can be adjusted by clicking on an intensity value in the histogram. All spots that currently pass the thresholding are displayed in the image window and marked by a red circle. If you are satisfied with the selected spots, press the **OK** button and continue to the final results table.

The **Log** window (**Fig. SN9.3b**) summarizes all spots found at every step and the final number of detections. The **Results table** (**Fig. SN9.3c**) contains the spot coordinates, time, channel, and intensity values in the corresponding columns. You can save the results and use them in the **Show Detections** plugin to visualize all found spots' locations.

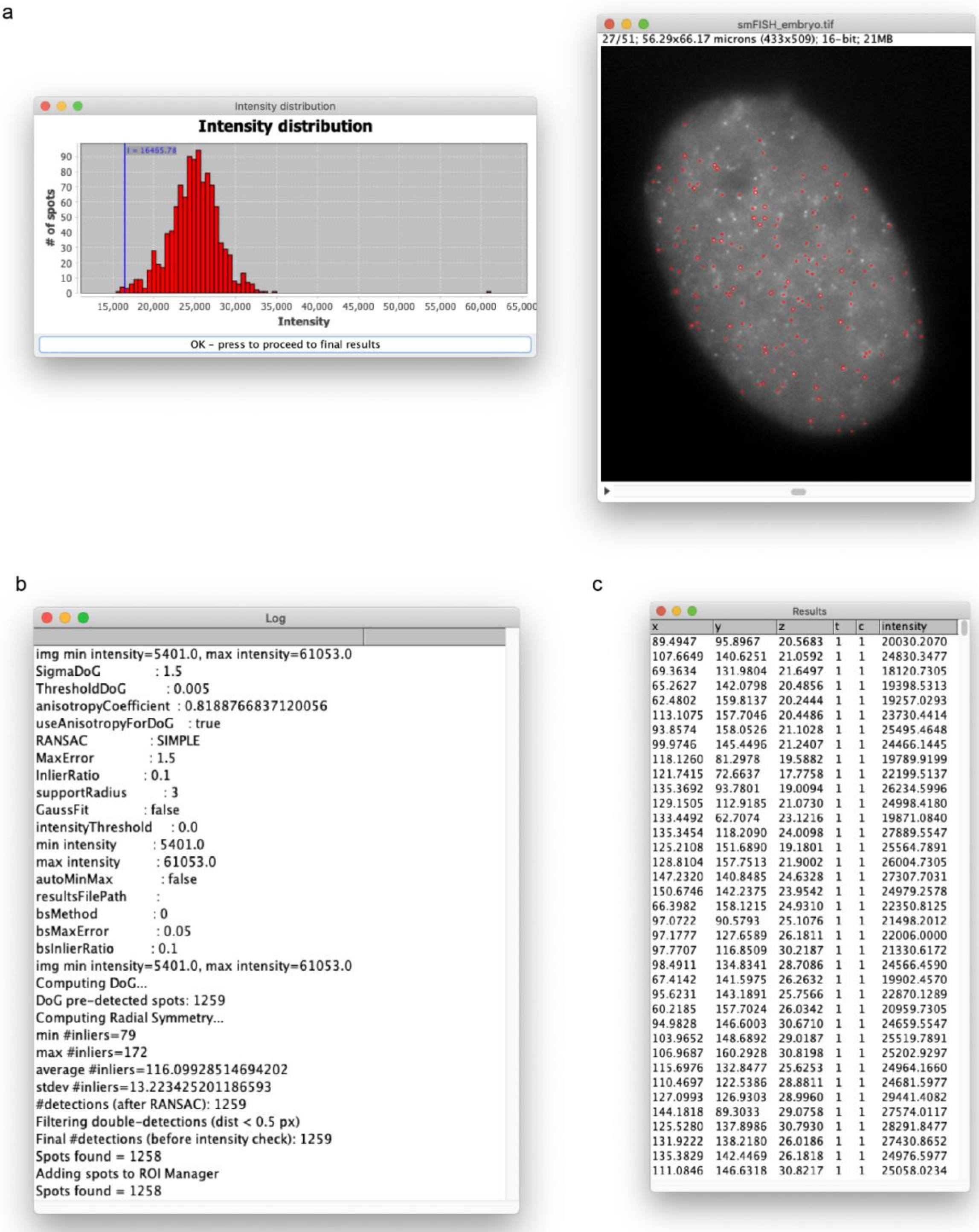

img min intensity=5401.0, max intensity=61053.0
SigmaDoG : 1.5
ThresholdDoG : 0.005
anisotropyCoefficient : 0.8188766837120056
useAnisotropyForDoG : true
RANSAC : SIMPLE
MaxError : 1.5
InlierRatio : 0.1
supportRadius : 3
GaussFit : false
intensityThreshold : 0.0
min intensity : 5401.0
max intensity : 61053.0
autoMinMax : false
resultsFilePath :
bsMethod : 0
bsMaxError : 0.05
bsInlierRatio : 0.1
img min intensity=5401.0, max intensity=61053.0
Computing DoG...
DoG pre-detected spots: 1259
Computing Radial Symmetry...
min #inliers=79
max #inliers=172
average #inliers=116.09928514694202
stdev #inliers=13.223425201186593
#detections (after RANSAC): 1259
Filtering double-detections (dist < 0.5 px)
Final #detections (before intensity check): 1259
Spots found = 1258
Adding spots to ROI Manager
Spots found = 1258

x	y	z	t	c	intensity
89.4947	95.8967	20.5683	1	1	20030.2070
107.6649	140.6251	21.0592	1	1	24830.3477
69.3634	131.9804	21.6497	1	1	18120.7305
65.2627	142.0798	20.4856	1	1	19398.5313
62.4802	159.8137	20.2444	1	1	19257.0293
113.1075	157.7046	20.4486	1	1	23730.4414
93.8574	158.0526	21.1028	1	1	25495.4648
99.9746	145.4496	21.2407	1	1	24466.1445
118.1260	81.2978	19.5882	1	1	19789.9199
121.7415	72.6637	17.7758	1	1	22199.5137
135.3692	93.7801	19.0094	1	1	26234.5996
129.1505	112.9185	21.0730	1	1	24998.4180
133.4492	62.7074	23.1216	1	1	19871.0840
135.3454	118.2090	24.0098	1	1	27889.5547
125.2108	151.6890	19.1801	1	1	25564.7891
128.8104	157.7513	21.9002	1	1	26004.7305
147.2320	140.8485	24.6328	1	1	27307.7031
150.6746	142.2375	23.9542	1	1	24979.2578
66.3982	158.1215	24.9310	1	1	22350.8125
97.0722	90.5793	25.1076	1	1	21498.2012
97.1777	127.6589	26.1811	1	1	22006.0000
97.7707	116.8509	30.2187	1	1	21330.6172
98.4911	134.8341	28.7086	1	1	24566.4590
67.4142	141.5975	26.2632	1	1	19902.4570
95.6231	143.1891	25.7566	1	1	22870.1289
60.2185	157.7024	26.0342	1	1	20959.7305
94.9828	146.6003	30.6710	1	1	24659.5547
103.9652	148.6892	29.0187	1	1	25519.7891
106.9687	160.2928	30.8198	1	1	25202.9297
115.6976	132.8477	25.6253	1	1	24964.1660
110.4697	122.5386	28.8811	1	1	24681.5977
127.0993	126.9303	28.9960	1	1	29441.4082
144.1818	89.3033	29.0758	1	1	27574.0117
125.5280	137.8986	30.7930	1	1	28291.8477
131.9222	138.2180	26.0186	1	1	27430.8652
135.3829	142.4469	26.1818	1	1	24976.5977
111.0846	146.6318	30.8217	1	1	25058.0234

Figure SN9.3: *Thresholding the detected spots before the final results table. a) Option of thresholding the detected spots based on their intensity by changing the blue Intensity (I) cut-off. Spots currently within the selected threshold are marked with red circles in the z-plane with the highest intensity. b) The Log window with selected parameters and the total number of spots found. c) The Results table for all detected spots with their x,y,z location, and intensity. RS-FISH uses 0.0, 0.0, 0.0 as the origin; i.e. each pixel is assumed to be a measurement located at a certain position, the first one being 0.0, 0.0, 0.0., and it outputs all locations in pixel values.*

Advanced mode

In the **Advanced mode**, you can skip the interactive setting of parameters and only use already known parameters for the computation. After choosing **Advanced** in the first window (**Fig. SN9.4a**), you will reach the second window to set the spot detection parameters (**Fig. SN9.4b**). If you previously used the interactive mode to find the best parameters, they will be saved and set as default for the advanced mode. After you press **OK,** the computation is automatically done in all RS-FISH steps, and the same **Results table** as above is either saved or displayed.

Scripting / headless

You can simply record the parameters you used for running RS-FISH (Plugins > Macro > Record). You can apply the found parameters to a set of images using the Fiji/ImageJ macro language.

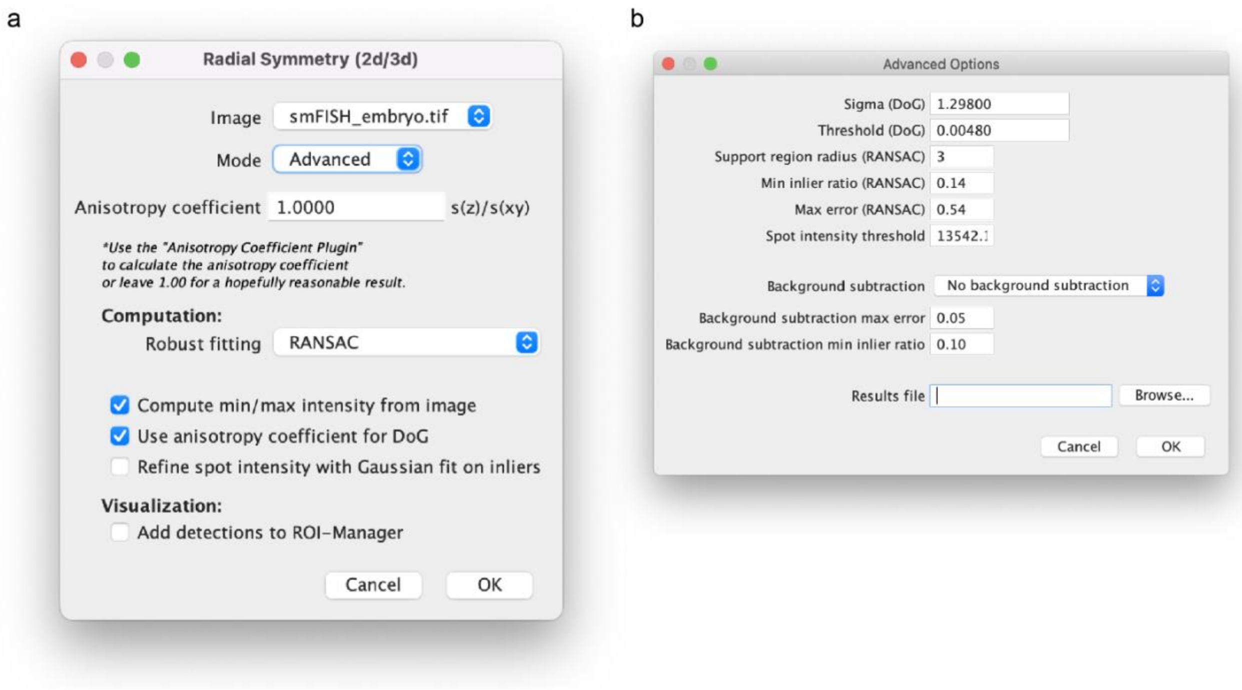

Figure SN9.4: *RS-FISH advanced mode. a) To run the advanced mode of RS-FISH, select "Advanced" as the running mode in the drop-down menu. b) The advanced options window allows you to select all detection parameters without manually adjusting them.*

Showing Detections

After RS-FISH computes all spots, the results table can be saved as CSV or directly be used to visualize the detected spots. There are three ways to visualize the RS-FISH detected spots:

- ROI Manager
- Fiji/ImageJ overlay
- BigDataViewer

If the option to transfer spots directly to the **ROI Manager** was chosen initially, the ROI manager would pop up with the results table in the last step (**Fig. SN9.5a**).

The **Show Detections (ImageJ/Fiji)** plugin (Plugins > RS-FISH > Tools > Show Detections (ImageJ/Fiji)) can be used to overlay all spots stored in a CSV onto the current image for visual inspection of the final result using Fiji (**Fig. SN9.5b**). The detected spots will be highlighted by red circles that are the largest in the z position where the center of the spot is.

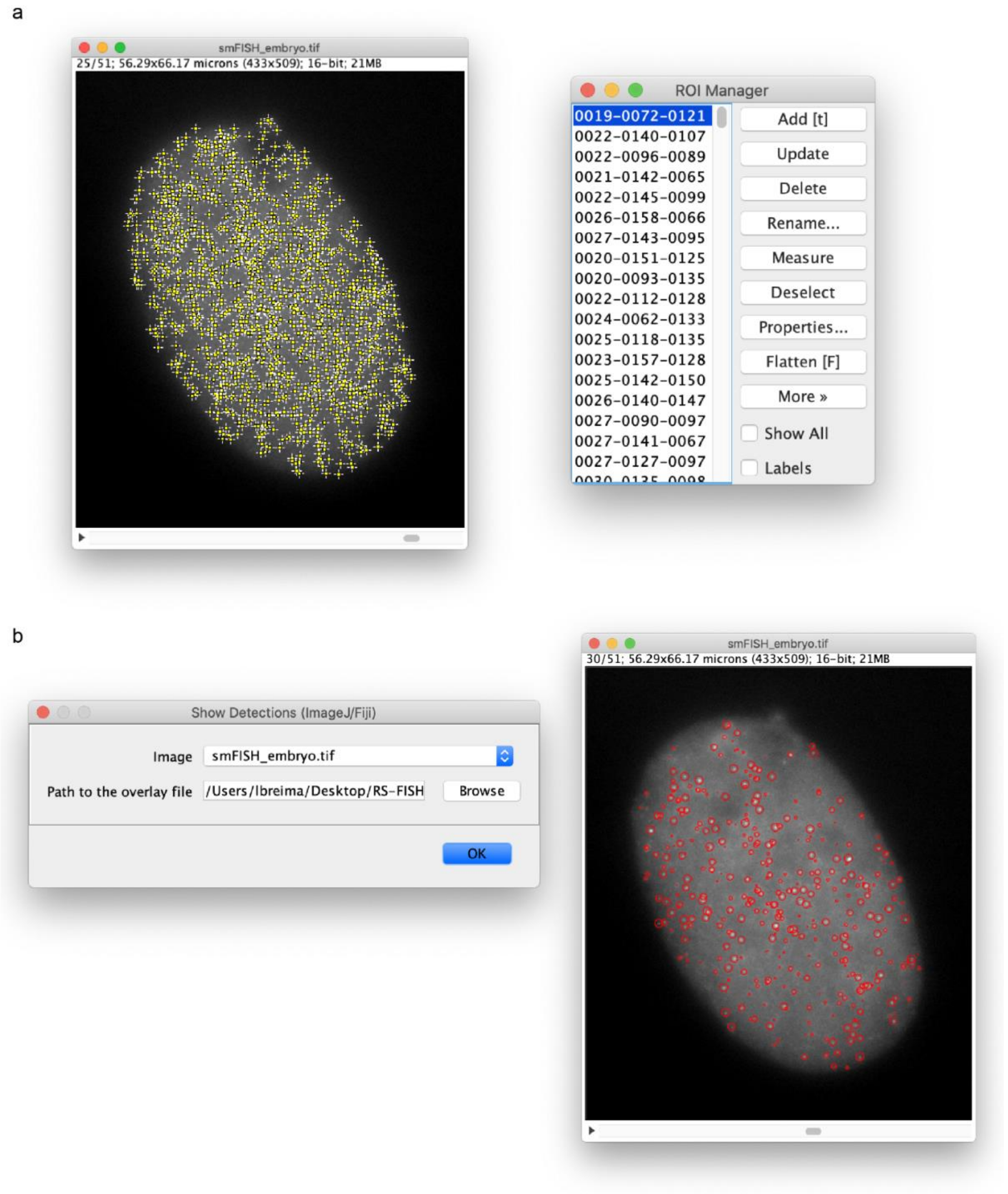

Figure SN9.5: *Visualization options for detected spots. a) The detections can be transferred to the ROI manager for visualization or selection of single spots. b) Show detections using the RS-FISH plugin for a previously processed image.*

The **Show Detections (BigDataViewer)** plugin (Plugins > RS-FISH > Tools > Show Detections (BigDataViewer)) can be used to visualize the spots using the BigDataViewer[8] (**Fig. SN9.6**). In the first window are four options whether to open a saved image or CSV or use the currently active image and table (**Fig. SN9.6a**). The intensity of the overlay points can be changed with the sigma slider.

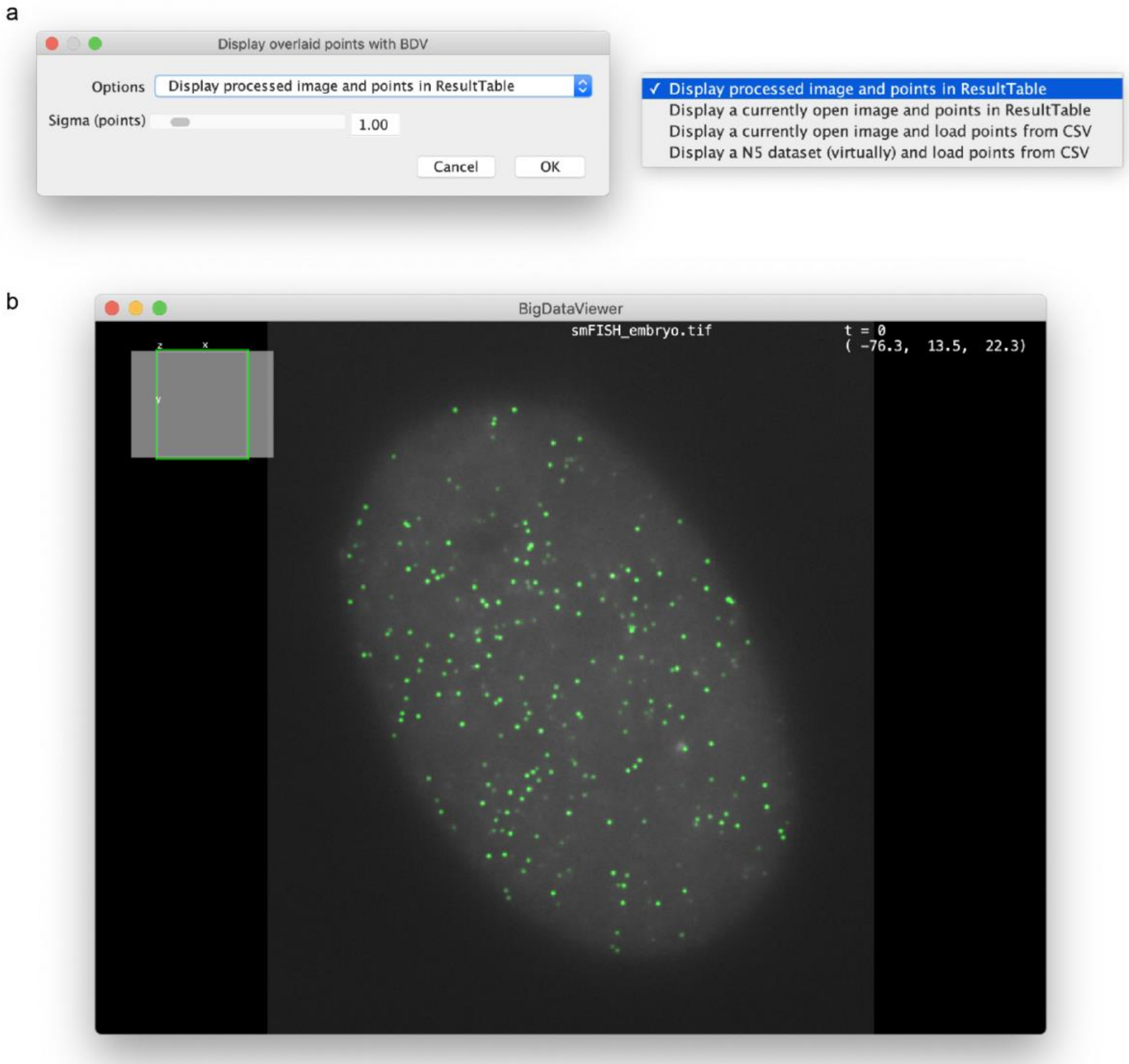

Figure SN9.6: *Show detections using the BigDataViewer***.** *a) Four options are available to display previously detected spots from either the open ResultsTable or a saved CSV. b) The BigDataViewer[8] displays detections (green) and the selected image (grayscale).*

10. Batch Processing and Macro Tutorial

RS-FISH plugin detections can also run from an ImageJ macro script from Fiji or headless from the terminal or a computing cluster.

This batch processing tutorial, as well as the example ImageJ macro script, can be found here:

https://github.com/PreibischLab/RS-FISH/tree/master/documents/example_scripts

A recommended procedure for running the plugin on a complete dataset is first running the plugin in Fiji in interactive mode on one (or a few) example images to find the best spot detection parameters. Then, use the parameters in a macro script that can be run from Fiji or headless.

Steps:
Find the best parameters for your dataset:

1. Open an example image from the dataset in Fiji.
2. Go to: Plugins > RS-FISH > RS-FISH
3. In the open menu, choose "Interactive" mode, set your image anisotropy coefficient (z resolution / XY resolution), you can leave the rest as default values (or change them according to the guide above), then click ok.
4. Change the slider values of the parameters until you are happy with the detections you see on the image (more info in the guide above). Then click “Done”.
5. You can also change the intensity threshold in the “Intensity distribution” window according to the detections seen on the image. Once the correct threshold has been set, click “OK - press to proceed to final results”.
6. Save the Fiji log, as it details the parameters used. For this, in the “Log” window, go to: File > Save As, and save the Log.txt file at your chosen location.

Run in batch mode:

1. Open the Log.txt file you just saved, so you can copy the parameters you found in interactive mode to the macro script.
2. Open the `RS_macro.ijm` (in Fiji or a text editor) from the GitHub link above.
3. In the macro file, change the parameters (e.g., `anisotropyCoefficient`, `sigmaDoG`) at the beginning of the macro file (under the line `Define RS parameter`) to the values from the Log.txt file. Unless you are sure otherwise, only change the values of existing variables in the macro file.
4. In the macro file, it is recommended to keep the `useMultithread` variable as “use_multithreading” for a speedier run. This option is not available when RS-FISH is run in “Interactive” mode. If multithreading is used, `numThreads` should be set according to the number of threads on your machine. `blockSizX`, `blockSizY`, and `blockSizZ` should be set for chunking each image in the analysis to blocks. Note, different multithreading runs may result in ever so slightly inconsistent results.
5. In the macro file, change the `path` variable value to the parent directory of your .tif images (all tifs in all subdirectories will be processed).
6. In the macro file, change the `timeFile` variable value to the path you wish to save the running times file.
7. Call the script. It can be done from the Fiji GUI, from the terminal, or from a computing cluster. Example Linux command to run the macro script:

    `<fiji_dir_path>/ImageJ-linux64 --headless --run </path/to/this/script>/RS_macro.ijm &> </path/to/where/you/want/yourlogfile>.log`

The macro above runs the same command as the command that is recorded when you record a run of the RS-FISH plugin in advanced mode.

Command Template:
`run("RS-FISH", "image=" + imName + " mode=Advanced anisotropy=" + anisotropyCoefficient + " robust_fitting=[" + ransacStr + "] use_anisotropy" + " image_min=" + imMin + " image_max=" + imMax + " sigma=" + sigmaDoG + " threshold=" + thresholdDoG + " support=" + supportRadius + " min_inlier_ratio=" + inlierRatio + " max_error=" + maxError + " spot_intensity_threshold=" + intensityThreshold + " background=[" + bsMethodStr + "] background_subtraction_max_error=" + bsMaxError + " background_subtraction_min_inlier_ratio=" + bsInlierRatio + " results_file=[" + results_csv_path + "]" + " " + useMultithreadStr + " num_threads=" + numThreads + " block_size_x=" + blockSizX + " block_size_y=" + blockSizY + " block_size_z=" + blockSizZ;`

Example command:
`run("RS-FISH", "image=im.tif mode=Advanced anisotropy=0.6500 robust_fitting=[RANSAC] use_anisotropy image_min=0 image_max=65535 sigma=1.203 threshold=0.0025 support=3 min_inlier_ratio=0.30 max_error=1.12237 spot_intensity_threshold=0 background=[No background subtraction] background_subtraction_max_error=0.05 background_subtraction_min_inlier_ratio=0.10 results_file=[/home/bob/Desktop/im_spots.csv] [use_multithreading] num_threads=40 block_size_x=128 block_size_y=128 block_size_z=16");`

Notably, running the tool/macro with a combination of parameters where sigma<1.5, threshold<0.002, and support>=3 will cause longer running times and requires bigger memory, especially for bigger images.

11. Distributed processing using RS-FISH-Spark

Distributed processing enables the analysis of terabyte-sized data or thousands of images. The Spark version of RS-FISH can analyze large N5 volumes in a distributed fashion locally, on the cluster, or in the cloud.

A tutorial on how to run a Spark-based version of RS-FISH can be found at https://github.com/PreibischLab/RS-FISH-Spark

12. Filtering detections using binary masks

To segment RS-FISH detections in different cells or subcellular structures like the nucleus, a binary mask of the region of interest can be used. These masks can be created manually using Fiji or segmentation tools like cellpose[11], ilastik[12], or stardist[13]. Notably, the binary mask should have a value of 0 for regions that are outside the structure of interest and have a value of 1 inside the structure.
The mask filtering tool can be found under 'Plugins' > 'RS-FISH' >’Tools’> 'Mask Filtering'. Selecting the Mask Filter tool opens a window to specify the input CSV and binary image mask file or folder **(Fig. -SN12.1a)**. If folders with several files are selected, files that belong together should be named similarly so the file names can be matched between result CSVs and binary mask files. The output of the mask filtering tool is a new CSV file, including only spots inside the binary mask **(Fig. SN12b)**.

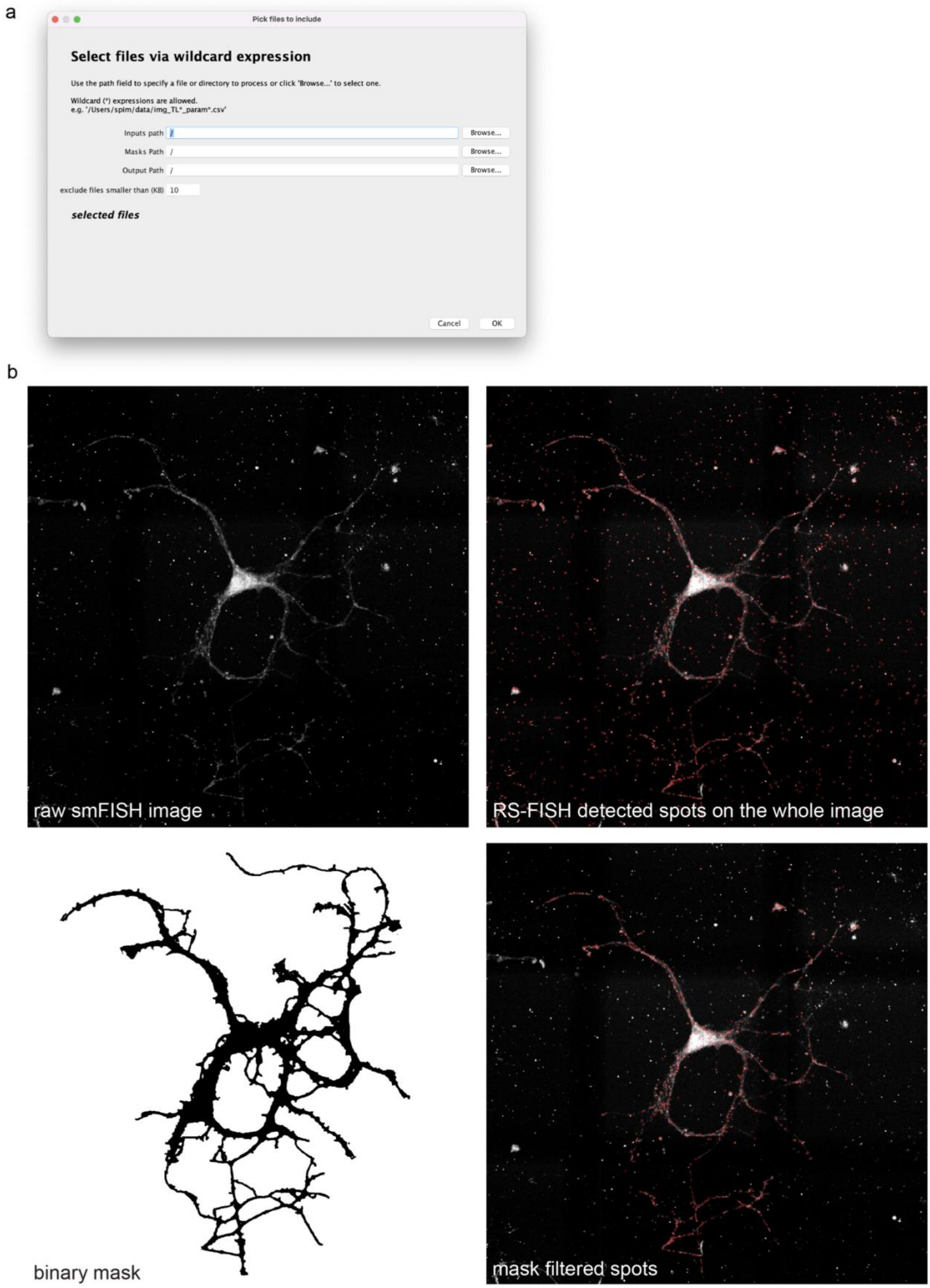

Figure SN12.1: *The mask filtering option in RS-FISH. (**a**) The mask filtering tool selects detections in the original image results table (input path) according to a binary file (mask path) and saves the new filtered table (output path). Files can be excluded according to their minimum size. (**b**) Example images of mouse primary cortical neurons to illustrate the mask filtering process. RS-FISH detects many spots in image (small red circles). To filter results, a binary mask was used in the mask filtering tool to allow detections only inside the central neuron (areas in black in the binary mask).The neuron image was kindly provided by Samantha Mendonsa and Marina Chekulaeva.*

13. smFISH protocol for *C. elegans* embryos and larvae

This protocol for *C. elegans* embryos was adapted from the Raj lab[14] protocol with some modifications.

Probe design

All used smFISH probe sets (Custom Stellaris® RNA FISH Probe Set) were designed and manufactured by Biosearch Technologies using the Stellaris probe designer. Probes were designed to target exons and labeled with either Quasar 670, CAL Fluor Red 610, or Quasar 570. Typically far-red dyes performed better due to the autofluorescence of *C. elegans* embryos, especially in older stages.

Preparation of worms for embryo collection

Worms were synchronized by either bleaching or egg-laying and grown to the adult stage. At this step, it is important to have sufficient amounts of non-starved, healthy adult worms. Typically at least 5 x 6-cm plates full of worms were used.

Collection of embryos

For embryo collection, worms were washed off plates using M9 and collected in a 35 µm nylon filter. Worms were washed at least three times with H2O, then carefully transferred to a falcon tube using M9 and allowed to settle down. The supernatant was removed, and 5 ml of freshly prepared bleaching solution was added to the worms. The dissolving of the adult worms was closely observed, and after 3-minutes, the tubes were spun at 3000 g for 1 min to collect the embryos. The supernatant was quickly removed, and the embryo pellet was vortexed. Then 10 ml of 1 x PBS-Triton were added, and the tube was centrifuged again for 3 min at 3000 g. This step was repeated two more times until a clean embryo extract was left in the tube.

Collection of larvae

For the collection of L1 larvae, worms were synchronized by either bleaching or egg-laying and grown to the adult stage. Embryos were collected by bleaching as described above, embryos were shaken in M9 overnight, and L1s were fixed the next morning.

Fixing and permeabilization

Embryos were resuspended in 1 ml fixation solution and incubated at RT for 15 min while rotating. Next, the tube was submerged into liquid nitrogen for 1 minute to freeze crack the embryo eggshells. The tube was then transferred to a beaker with RT water to thaw, and once it was fully thawed, it was kept on ice for an additional 20 minutes. After this incubation, the tubes were spun down at 3000 g for 3-minutes, and the supernatant was removed, and the embryos were washed twice with 1 ml of 1x PBS-Triton. The embryos were resuspended in 70% EtOH and kept at 4 °C for at least 24 hours. Embryos can be kept at 4 °C for at least several months.

smFISH staining

For smFISH staining of the previously fixed embryos, tubes were centrifuged at 3000 g for 3 minutes, and ethanol was carefully removed. The pellet can be pretty loose at this step, so removing the supernatant can also be done in two stages. Embryos were then resuspended in 1 ml wash buffer and vortexed. Tubes were centrifuged, as above, and the supernatant was removed. The embryos were resuspended in a 50 µl hybridization solution, and 1 µl of each probe set (12.5 µM stock solution) was added directly to the sample. Tubes were then vortexed lightly and incubated at 37°C in the dark overnight. The next day, 0.5 ml of the wash buffer was added, the tubes vortexed and centrifuged to remove the supernatant. Next, 1 ml of wash buffer was added, and samples were incubated at 37°C for 30 minutes. After that, tubes were again centrifuged to remove the supernatant, and the embryo pellet

was vortexed before adding 1 ml wash buffer. In this step, DAPI (5 ng/mL) was added to the wash buffer, and tubes were incubated at 37 °C for 30 minutes. After centrifugation, the wash buffer was removed, and samples were washed once with 2x SSC.

Mounting

To mount the stained embryos, most of the liquid was removed from the tubes. About 15 µl of dense embryos (in 2x SSC) were used per glass slide and spread onto a coverslip (#1.5, 22 x 22 mm). The sample was left to dry for about 15 minutes, and then 15 µl of ProLong™ Diamond Antifade Mountant (Thermo Fisher) was added to the sample. The glass slide (SuperFrost, VWR) was pressed with the embryos and mounting media onto the coverslip. Slides were left at RT in the dark 24 hours before sealing the sides with nail polish and then stored at 4°C. Images were then acquired within two weeks of preparing the sample.

Imaging

Embryos were imaged on a Nikon Ti inverted fluorescence microscope with an EMCCD camera (ANDOR, DU iXON Ultra 888), Lumen 200 Fluorescence Illumination Systems (Prior Scientific), and a 100x plan apo objective (NA 1.4) using appropriate filter sets. Images were acquired with 90 z-stacks positions with 200 nm step-width using Nikon Elements software.

Buffer

- Collection buffer M9: 5.8 g Na_2HPO_4, 3.0 g KH_2PO_4, 0.5 g NaCl, 1.0 g NH_4Cl, Nuclease-free water to a final volume of 1000 ml
- 1x PBS (DEPC treated + autoclave + 0,05% of Triton X-100)
- Fixing buffer: 4% paraformaldehyde (PFA) in 1xPBS (DEPC treated + autoclave + 0,05% of Triton X-100)
- Bleaching stock solution: 2.5 ml 4N NaOH, 2.5 ml 5% NaClO, 5 ml Nuclease-free water, freshly made
- Washing buffer: 40 ml nuclease-free water, 5 ml deionized formamide, 5 ml 20x SSC
- Hybridisation solution: 50 µl H20 (RNAse free), 37.5 µl EC 5 mg/ml, 25 µl formamide (at RT), 12.5 µl SDS (dissolved), 125 µl dextran sulphate 10 %

14. smFISH protocol for mouse ES cells using cytospin

Cell culture

All pipetting steps were carried out at room temperature and extra care was taken never to let cells dry between buffer changes. Cells were grown in a 10 cm dish to 70-80% confluency and harvested by adding 1 ml warm trypsin. After 10 min, cells were carefully resuspended by adding 1 ml of warm ESC medium (DMEM (high glucose, without sodium pyruvate, cat no 41965062, Gibco, Life Technologies Ltd, UK), supplemented with 15% FCS, 100 µg/ml penicillin/streptomycin, 2 mM L-Glutamine (Gibco, UK), 1x Non-essential amino acids (MEM-NEAA) (Gibco, UK), 50 µM beta-mercaptoethanol (Gibco, UK) and 10 ng/ml leukemia inhibitory factor (LIF, prepared in house)) and pipetting up and down several times. Cells were collected in a falcon tube with 4 ml ESC-medium and centrifuged at 1000 rpm on a Heraeus Multifuge L3 for 3 min (room temperature).

Single-molecule FISH

Cells were resuspended in 5 ml warm PBS, counted using a Neubauer chamber, and 1 ml of 4-6x10^5 cells per ml were transferred to a new falcon tube. To fix the cells, an equal volume of fixing solution (5-ml 10x PBS, 5 ml 37% formaldehyde, 40 ml H2O, in sample 2% PFA final concentration) and incubated for exactly 10 min at RT. Per slide, 100 µl of the solution was pipetted in an assembled cytofunnel (Shandon Single Cytofunnel, Thermo Fisher Scientific) and centrifuged in a Cytospin 4 (Shandon Cytospin, Thermo Fisher Scientific) at 1800 rpm for 3 min (with high acceleration setting). Cytofunnels were removed quickly, and slides were washed in 1x PBS for 5 min at room temperature. To permeabilize the cells, slides were immersed in 70% ethanol and incubated at 4°C for 1 hour. Slides were washed in a washing buffer (5 ml 20x SSC, 5 ml formamide, 40 ml H2O) for 5 min at room temperature. During this step, the humidified chamber was assembled. A plastic container was lined with wet paper towels, and a slide holder was placed on top. To 50 µl room temperature, warm hybridization solution (100 mg/ml dextran sulfate and 10% formamide in 2X SSC) 0.5 µl of probe stock solution (12.5 µM) were added, mixed by vortexing, and collected by a short centrifugation step. This resulted in a final probe concentration of 125 nM. A 50 µl drop of the probe containing hybridization buffer was added to the surface of a coverslip of the area where the cells were fixed. Slides were placed in the humidified chamber and incubated in the dark for 4 hours at 37°C.
Gently, slides were transferred to a fresh beaker containing a wash buffer after removing the coverslip. This washing step continued for 30 min in the dark at 37°C. Afterward, the washing buffer was replaced by a DAPI-containing washing buffer (5 ng/ml) and incubated again in the dark at 37 °C for 30 minutes. The DAPI staining buffer was collected, and the final buffer (2 X SSC) was added and incubated at room temperature for 5 minutes. Slides were mounted with 25 µl of Vectashield Mounting Medium, which was pipetted using a cut 200µl pipet tip. Excess fluid from the perimeter of the coverglass was gently wiped away, and the cover glass was sealed with clear nail polish to preserve the slides for a longer time. After 10 minutes of drying, slides were stored in a light-tight box at 4°C until being used for imaging.

Image acquisition

Slides were imaged on a wide-field fluorescence microscope (DeltaVision Elite) using a 63x/1.4 N.A. plan-apochromat oil-immersion objective with N=1.514 oil (Applied Precision). The slides were illuminated with an SSI - LED light at different intensities. An Evolve EMCCD camera was used for image acquisition.

15. smFISH protocol for cleared whole-mount adult Drosophila brains

A detailed description of the FISH method, and FISH probe design was described previously (Long et al., 2017[15]). Briefly, 3-5-day-old adult *Drosophila* were dissected in PBS and fixed for 55 min at RT in 2% paraformaldehyde. The tissues were then dehydrated and stored in 100% ethanol overnight at 4°C.

After exposure to 5% acetic acid at 4°C for 5 min, the tissues were fixed again. The tissues were then incubated in PBS with 1% $NaBH_4$ at 4°C for 30 min, followed by a 2 h incubation in prehybridization buffer at 50°C (15% formamide, 2 × SSC and 0.1% Triton X-100). The tissues were transferred to 50-μL of hybridization buffer (10% formamide, 2 × SSC, 5 × Denhardt’s solution, 1 mg/ml yeast tRNA, 100 μg/ml salmon sperm DNA, 0.1% SDS) with FISH probes against the targeted transcripts (50–100-ng/μL per reaction) and incubated at 50°C for 10 h and then at 37°C for an additional 10 h. After several wash steps, the tissues were dehydrated and cleared by xylene. The cleared tissues were mounted on a 1.5x3 mm coverslip attached to the end of a 30 mm glass rod and imaged in a mixture of 90% 1,2-dichlorobenzene and 10% 1,2,4-trichlorobenzene medium.

16. smFISH protocol for Kuramochi cells

Cell culture

Kuramochi cells[10] were cultured in RPMI 1640 media (ATCC 30-2001) supplemented with 10% FBS (VWR Seradigm 97068085), 1% L-glutamine (Gibco 25030081), 1% antibiotic/antimycotic (Gibco 15240062), 0.25 U/ml recombinant human insulin (Sigma I9278), and 1x MEM non-essential amino acids (Gibco 11140050) at 37°C with 5% CO_2. Cells were routinely passaged using Trypsin 0.25% EDTA dissociation reagent (Gibco 25200056).

Single-molecule FISH

mRNAs were labeled for individual molecule detection using a previously described protocol[16]. Kuramochi cells were grown on 18 mm coverslips, rinsed with PBS, and fixed with 4%-paraformaldehyde in PBS for 10 min at room temperature (RT). Coverslips were rinsed with PBS, and permeabilized with PBS containing 0.5% Triton X 100 for 10 min. Coverslips were washed with PBS and pre-hybridized for 10 min in 10% deionized formamide in 2x sodium chloride sodium citrate (SSC) before hybridization overnight at 37°C. Hybridization buffer contains 10% formamide, 0.2 mg/ml *E. coli* tRNA, 0.2 mg/ml salmon sperm DNA, 10% dextran sulfate, 2 mg/ml BSA, and 10 ng of 20mer smFISH probes targeting *IGF2 mRNA* covalently labeled with Cy5 fluorescent dye. After overnight hybridization, cells were washed with 10% formamide in 2x SSC for 20 min at 37°C. Coverslips were washed for 20 min at RT with 10% formamide in 2x SSC on a slow shaker and washed for 10 min at RT with PBS. Coverslips were incubated for 1 min with DAPI (0.5 mg/L in PBS) to counterstain DNA. Final washes were completed with PBS twice for 5 min each before mounting coverslips on slides using Prolong Gold antifade reagent (Invitrogen).

Image acquisition

Images were acquired on a Nikon inverted Ti-E eclipse microscope equipped for wide-field epifluorescence. Fluorescence was excited using one of two lasers (405 nm Vortran, 637 nm Vortran), a 100x Plan Apo NA 1.4 oil immersion objective, and an Andor Ixon EM-CCD. Hardware was controlled and synchronized using Micro-Manager[17]. Lasers were shuttered digitally using TTL modulation. Individual images consist of 16-bit gray level, 512x512 pixels (lateral pixel dimension in sample plane: 161 nm). smFISH images were collected across three power levels and exposure times to capture a range of spot intensities - 2.5 V (70 mW) for 10 ms, 5 V (160 mW) for 20 ms, and 5 V (160 mW) for 100-ms.

IGF2 probe sequences:

Probe#	sequence	Position	%GC
1	acagagaagcggagggaagg	555	60.00%
2	attaatccactttggttcgg	710	40.00%
3	ggagagaacagcacggagag	746	60.00%
4	ggtatcgggaaatgaggtca	889	50.00%
5	agatgttgtacttttcgggg	916	45.00%
6	gaattcgtctgattgtccag	969	45.00%
7	cacagacgaactggagggtg	1284	60.00%
8	gaaacagcactcctcaacga	1364	50.00%
9	ccaggtgtcatattggaaga	1505	45.00%
10	gaacctgatggaaacgtccg	1769	55.00%
11	gacgtggaaccgagagattt	1797	50.00%
12	tgtttgaagatgctgctgtg	1905	45.00%
13	gggtgtttaaagccaatcga	1934	45.00%
14	ataatttggggggagggtat	1959	45.00%
15	ttaagagggttgttgtgggg	2036	50.00%

16	ccaaattcctttattttgcc	2182	35.00%
17	gccaatttgactcaaagtcc	2234	45.00%
18	agtttaccctgaaaattccc	2346	40.00%
19	ggttgttgctattttcggat	2370	40.00%
20	aaaagggagtgaggagccag	2394	55.00%
21	ctgactgctctgtgattttt	2422	40.00%
22	tgtaagatttgggggtgagg	2522	50.00%
23	tgagtgctttttaggatggg	2550	45.00%
24	atgcttattgttttcatcca	3287	30.00%
25	cctgatccatacagatatcg	3320	45.00%
26	gagaatcttagcgggacttt	3342	45.00%
27	gggctcagaccatgaaaaca	3365	50.00%
28	cagtggagatgggaacagga	3388	55.00%
29	aagatggaattaggcctggg	3519	50.00%
30	aaggtgtatcgggaatgggt	3632	50.00%
31	caaagatgatccctaggtgt	3798	45.00%
32	cctgacttttccatccaaaa	3970	40.00%
33	aattctgcaagtctgctgtc	3996	45.00%
34	aatttggtttctgagcgcat	4136	40.00%
35	ctcaactcaacccaagcaac	4284	50.00%
36	tgatggaaccctctgtttac	4540	45.00%
37	tctgtccaatgttccgaatg	5047	45.00%
38	gggttccacaattgtgacaa	5083	45.00%
39	cgaagcgttttggatctcag	5111	50.00%
40	gaatgggcaggtaatttggg	5137	50.00%
41	ccggcgaggcagaatataac	5180	55.00%
42	gagacaagatggagagccac	5317	55.00%
43	tgaagacattggggacacgg	5341	55.00%
44	cacaggtgacattcagtgtt	5418	45.00%
45	gagttgagtcaaacacgggc	5462	55.00%
46	ttgccggaaatattagcgtt	5491	40.00%
47	gacaaaacccaagcatggga	5513	50.00%
48	attgggattgcaagcgttac	5542	45.00%

References

1. Parthasarathy, R. Rapid, accurate particle tracking by calculation of radial symmetry centers. *Nature Methods* 9, 724–726 (2012).

2. Press, W. H., Flannery, B. P., Teukolsky, S. A. & Vetterling, W. T. Numerical Recipes: The Art of Scientific Computing. *Comput Geosci* 15, 1199–1200 (1990).

3. Mueller, F. *et al.* FISH-quant: automatic counting of transcripts in 3D FISH images. *Nature Methods* 10, 277–278 (2013).

4. Imbert, A. *et al.* FISH-quant v2: a scalable and modular tool for smFISH image analysis. *Rna* 28, rna.079073.121 (2022).

5. Lionnet, T. *et al.* A transgenic mouse for in vivo detection of endogenous labeled mRNA. *Nat Methods* 8, 165–170 (2011).

6. S, A. *et al.* Starfish: Open Source Image Based Transcriptomics and Proteomics Tools. (2018).

7. Eichenberger, B. T., Zhan, Y., Rempfler, M., Giorgetti, L. & Chao, J. A. deepBlink: threshold-independent detection and localization of diffraction-limited spots. *Nucleic Acids Res* 49, 7292–7297 (2021).

8. Nguyen, H. Q. *et al.* 3D mapping and accelerated super-resolution imaging of the human genome using in situ sequencing. *Nat Methods* 17, 822–832 (2020).

9. Wang, Y. *et al.* EASI-FISH for thick tissue defines lateral hypothalamus spatio-molecular organization. *Cell* 184, 6361-6377.e24 (2021).

10. [Biological characterization including sensitivity to mitomycin C of cultured human ovarian cancers (author's transl)] - PubMed. https://pubmed.ncbi.nlm.nih.gov/6268719/.

11. Stringer, C., Wang, T., Michaelos, M. & Pachitariu, M. Cellpose: a generalist algorithm for cellular segmentation. *Nat Methods* 18, 100–106 (2021).

12. Berg, S. *et al.* ilastik: interactive machine learning for (bio)image analysis. *Nat Methods* 16, 1226–1232 (2019).

13. Schmidt, U., Weigert, M., Broaddus, C. & Myers, G. Cell Detection with Star-convex Polygons. *Arxiv* (2018) doi:10.1007/978-3-030-00934-2_30.

14. Raj, A. & Tyagi, S. Chapter 17 Detection of Individual Endogenous RNA Transcripts In Situ Using Multiple Singly Labeled Probes. *Methods Enzymol* 472, 365–386 (2010).

15. Long, X., Colonell, J., Wong, A. M., Singer, R. H. & Lionnet, T. Quantitative mRNA imaging throughout the entire Drosophila brain. *Nat Methods* 14, 703–706 (2017).

16. Raj, A., Bogaard, P. van den, Rifkin, S. A., Oudenaarden, A. van & Tyagi, S. Imaging individual mRNA molecules using multiple singly labeled probes. *Nature Publishing Group* 5, 877–879 (2008).

17. Edelstein, A. D. *et al.* Advanced methods of microscope control using μManager software. *J Biological Methods* 1, e10 (2014).
