# Supplementary material for: RS-FISH: precise, interactive, fast, and scalable FISH spot detection
Source: Nat Methods. 2022 Nov 17;19(12):1563–7. doi: 10.1038/s41592-022-01669-y (PMC9718671; doi:10.1038/s41592-022-01669-y)
Supplement: Supplementary file 2 — Reporting Summary [file 41592_2022_1669_MOESM2_ESM.pdf]

nature research

Corresponding author(s): K. Harrington, T. Lionnet, S. Preibisch

Last updated by author(s): Sep 7, 2022

Reporting Summary

Nature Research wishes to improve the reproducibility of the work that we publish. This form provides structure for consistency and transparency in reporting. For further information on Nature Research policies, see our Editorial Policies and the Editorial Policy Checklist.

Statistics

For all statistical analyses, confirm that the following items are present in the figure legend, table legend, main text, or Methods section.

n/a	Confirmed	
☐	☒	The exact sample size (*n*) for each experimental group/condition, given as a discrete number and unit of measurement
☐	☒	A statement on whether measurements were taken from distinct samples or whether the same sample was measured repeatedly
☒	☐	The statistical test(s) used AND whether they are one- or two-sided<br>*Only common tests should be described solely by name; describe more complex techniques in the Methods section.*
☒	☐	A description of all covariates tested
☒	☐	A description of any assumptions or corrections, such as tests of normality and adjustment for multiple comparisons
☐	☒	A full description of the statistical parameters including central tendency (e.g. means) or other basic estimates (e.g. regression coefficient) AND variation (e.g. standard deviation) or associated estimates of uncertainty (e.g. confidence intervals)
☒	☐	For null hypothesis testing, the test statistic (e.g. *F*, *t*, *r*) with confidence intervals, effect sizes, degrees of freedom and *P* value noted<br>*Give P values as exact values whenever suitable.*
☒	☐	For Bayesian analysis, information on the choice of priors and Markov chain Monte Carlo settings
☒	☐	For hierarchical and complex designs, identification of the appropriate level for tests and full reporting of outcomes
☒	☐	Estimates of effect sizes (e.g. Cohen's *d*, Pearson's *r*), indicating how they were calculated

Our web collection on statistics for biologists contains articles on many of the points above.

Software and code

Policy information about availability of computer code

Data collection	Micro-Manager (2.0.0), NIS Elements software, Applied Precision SoftWoRx
Data analysis	RS-FISH (0f6ab4a), FISH-quant (v3), Big-FISH (0.5.0), AIRLOCALIZE (1.6), Starfish (0.2.2), deepBlink (0.1.1), Fiji (2.3.0)

For manuscripts utilizing custom algorithms or software that are central to the research but not yet described in published literature, software must be made available to editors and reviewers. We strongly encourage code deposition in a community repository (e.g. GitHub). See the Nature Research guidelines for submitting code & software for further information.

Data

Policy information about availability of data

All manuscripts must include a data availability statement. This statement should provide the following information, where applicable:

- Accession codes, unique identifiers, or web links for publicly available datasets
- A list of figures that have associated raw data
- A description of any restrictions on data availability

All datasets used for benchmarking are available in the RS-FISH GitHub repository (https://github.com/PreibischLab/RS-FISH and https://github.com/timotheelionnet/simulated_spots_rsFISH), which includes simulations and 3D smFISH images of C. elegans embryos. The raw data underlying Fig. 1 are available at figshare: https://doi.org/10.6084/m9.figshare.21067342.v1; https://doi.org/10.6084/m9.figshare.21067366; https://doi.org/10.6084/m9.figshare.21067360.v1; https://doi.org/10.6084/m9.figshare.21067354; https://doi.org/10.6084/m9.figshare.21067369.v1; https://doi.org/10.6084/m9.figshare.21067372

Field-specific reporting

Please select the one below that is the best fit for your research. If you are not sure, read the appropriate sections before making your selection.

☒ Life sciences ☐ Behavioural & social sciences ☐ Ecological, evolutionary & environmental sciences

For a reference copy of the document with all sections, see nature.com/documents/nr-reporting-summary-flat.pdf

Life sciences study design

All studies must disclose on these points even when the disclosure is negative.

Sample size	No measure were taken to estimate the sample sizes. However the sample size was limited by the computational capacity since we performed grid searches over the parameter space for each tool and analysis. The sample size was adequate as it illustrates the detection capabilities of RS-FISH compared to similar tools. The sample size of 50 images for localization performance were chosen to cover a reasonable range of simulated SNRs and point densities (each containing 30-300 points) - all can be inspected in the github repository. For the analysis of close points we created 720 images (each containing 30 points). For the benchmarks using real data we created 63 two-dimensional images with different noise levels. For speed measurements we chose 13 different samples of actual 3D image data stacks. We limited it to 13 since each tool needs to be manually tuned to yield similar numbers of points, which constitutes a major effort.
Data exclusions	Data in Supplementary Note 6 was excluded due to too low SNR (explained in the text). Otherwise no data was excluded.
Replication	Unless stated otherwise, experiments were performed once. The manuscript asses the performance of an image analysis software (mostly on simulated data) and not on variability of biological samples.
Randomization	The location of the points for the benchmarking datasets were chosen randomly. Data analysis was not randomized since this analysis is not affected by human bias.
Blinding	The benchmarking was performed and scored by computer algorithms after a grid search to find the best parameters for each tool. Since this analysis is not affected by human bias, no blinding was necessary.

Reporting for specific materials, systems and methods

We require information from authors about some types of materials, experimental systems and methods used in many studies. Here, indicate whether each material, system or method listed is relevant to your study. If you are not sure if a list item applies to your research, read the appropriate section before selecting a response.

Materials & experimental systems

n/a	Involved in the study	
☒	☐	Antibodies
☐	☒	Eukaryotic cell lines
☒	☐	Palaeontology and archaeology
☐	☒	Animals and other organisms
☒	☐	Human research participants
☒	☐	Clinical data
☒	☐	Dual use research of concern

Methods

n/a	Involved in the study	
☒	☐	ChIP-seq
☒	☐	Flow cytometry
☒	☐	MRI-based neuroimaging

Eukaryotic cell lines

Policy information about cell lines

Cell line source(s)	Cell lines were obtained from JCRB Cell Bank (JCRB0098 KURAMOCHI)
Authentication	As cell lines were purchased directly from JCRB, visual inspection was used to confirm morphology was consistent with description and pictures provided by the vendor.
Mycoplasma contamination	Cells lines tested negative for mycoplasma contamination
Commonly misidentified lines (See ICLAC register)	No commonly misidentified cell lines were used in the study

Animals and other organisms

Policy information about studies involving animals; ARRIVE guidelines recommended for reporting animal research

Laboratory animals	C. elegans: N2 wild-type worms, hermaphrodite, stages mixed embryo and L2 larvae; Drosophila: strain: wild-type (w1118) ; sex: female ; stage: ZT2
Wild animals	No wild animals were used in this study.
Field-collected samples	No field-collected samples were used in this study.
Ethics oversight	No ethical approval was required for Drosophila and C. elegans

Note that full information on the approval of the study protocol must also be provided in the manuscript.
